# Complex Inhibitory Effects of Nitric Oxide on Autophagy

**DOI:** 10.1016/j.molcel.2011.04.029

**Published:** 2011-07-08

**Authors:** Sovan Sarkar, Viktor I. Korolchuk, Maurizio Renna, Sara Imarisio, Angeleen Fleming, Andrea Williams, Moises Garcia-Arencibia, Claudia Rose, Shouqing Luo, Benjamin R. Underwood, Guido Kroemer, Cahir J. O'Kane, David C. Rubinsztein

**Affiliations:** 1 Department of Medical Genetics, University of Cambridge, Cambridge Institute for Medical Research, Addenbrooke's Hospital, Hills Road, Cambridge, CB2 0XY, UK; 2 Department of Genetics, University of Cambridge, Downing Street, Cambridge CB2 3EG, UK; 3 Department of Physiology, Development and Neuroscience, University of Cambridge, Downing Street, Cambridge CB2 3EG, UK; 4 INSERM, U848, Villejuif F-94805, France; 5 Metabolomics Platform, Institut Gustave Roussy, 94805 Villejuif, France; 6 Centre de Recherche des Cordeliers, 75006 Paris, France; 7 Pôle de Biologie, Hôpital Européen Georges Pompidou, AP-HP, 75015 Paris, France; 8 Université Paris Descartes, Paris 5, Paris, France

## Abstract

Autophagy, a major degradation process for long-lived and aggregate-prone proteins, affects various human processes, such as development, immunity, cancer, and neurodegeneration. Several autophagy regulators have been identified in recent years. Here we show that nitric oxide (NO), a potent cellular messenger, inhibits autophagosome synthesis via a number of mechanisms. NO impairs autophagy by inhibiting the activity of *S*-nitrosylation substrates, JNK1 and IKKβ. Inhibition of JNK1 by NO reduces Bcl-2 phosphorylation and increases the Bcl-2–Beclin 1 interaction, thereby disrupting hVps34/Beclin 1 complex formation. Additionally, NO inhibits IKKβ and reduces AMPK phosphorylation, leading to mTORC1 activation via TSC2. Overexpression of nNOS, iNOS, or eNOS impairs autophagosome formation primarily via the JNK1–Bcl-2 pathway. Conversely, NOS inhibition enhances the clearance of autophagic substrates and reduces neurodegeneration in models of Huntington's disease. Our data suggest that nitrosative stress-mediated protein aggregation in neurodegenerative diseases may be, in part, due to autophagy inhibition.

## Graphical abstract

**Graphical Abstract:**

**Figure:**
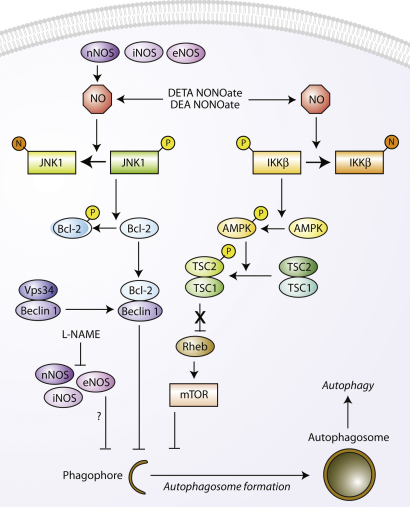

**Highlights:**

► NO inhibits autophagy by independently inhibiting JNK1 and IKKβ ► NO inhibits autophagic flux via mTOR and mTOR-independent routes ► NOS overexpression impairs autophagosome synthesis via JNK1–Bcl-2 pathway ► NOS inhibition induces autophagy and protects against neurodegeneration

## Introduction

Macroautophagy (henceforth referred to as “autophagy”) is an intracellular bulk degradation process involved in the clearance of long-lived proteins and organelles, which affects various physiological and pathological processes, including development, immunity, longevity, cancer, and neurodegenerative diseases (Mizushima et al., 2008). Autophagy initiates with the formation of isolation membranes called phagophores, which elongate and engulf a portion of the cytoplasm to form mature autophagosomes. Autophagosomes fuse with lysosomes to form autolysosomes, in which acidic lysosomal hydrolases degrade the engulfed contents. Autophagy is a highly conserved process; genetic analyses in yeast have identified several *ATG* (AuTophaGy related) genes, many of which have mammalian orthologs. The only known mammalian protein that specifically associates with autophagosome membranes throughout their lifespan is microtubule-associated protein 1 (MAP1) light chain 3 (LC3), the mammalian ortholog of yeast Atg8. LC3 normally exists in the cytosol as LC3-I. Upon autophagy induction, LC3-I conjugates with phosphatidylethanolamine to form the autophagosome-associated LC3-II. LC3-II levels (relative to actin/tubulin loading controls) correlate with autophagosome numbers (Kabeya et al., 2000).

The classical pathway regulating mammalian autophagy involves the serine/threonine kinase, mammalian target of rapamycin (mTOR) (Rubinsztein et al., 2007). This protein negatively regulates autophagy via the mTOR complex 1 (mTORC1), which consists of raptor, GβL, and PRAS40 (Guertin and Sabatini, 2009). The activity of mTORC1 can be inhibited by rapamycin or starvation, which are well-established inducers of autophagy (Noda and Ohsumi, 1998). Many diverse signals, such as growth factors, amino acids, and energy status, regulate autophagy by the mTORC1 pathway (Meijer and Codogno, 2006). Recent studies have shown that mTORC1 regulates autophagy by acting on a complex comprising mammalian Atg13, ULK1, and FIP200 (Mizushima, 2010).

Starvation also regulates autophagy by activating c-Jun N-terminal kinase 1 (JNK1), which in turn phosphorylates Bcl-2 at multiple sites (T69, S70, and S87). Bcl-2 normally inhibits autophagy by interacting with the autophagy protein Beclin 1, whereas Bcl-2 phosphorylation inhibits this interaction to stimulate autophagy (Pattingre et al., 2005; Wei et al., 2008). Beclin 1 is a part of the class III phosphatidylinositol 3-kinase (PI3K)/hVps34 complex, and its binding with hVps34 is essential for the initiation of autophagosome formation (Pattingre et al., 2008).

Autophagy can also be regulated independently of mTOR (Sarkar et al., 2009b). Recent work has shown IKK (IκB kinase) to be an important regulator of autophagy induction via multiple stimuli. IKK regulates autophagy independently of its effects on NFκB and acts both by enhancing AMPK phosphorylation-dependent mTOR inhibition and JNK1-mediated Bcl-2 phosphorylation (Criollo et al., 2010).

Reactive oxygen species (ROS) also regulate autophagy under amino acid and serum starvation conditions, where superoxide has been suggested to be the major ROS species involved in ROS-mediated autophagy (Chen et al., 2009; Scherz-Shouval et al., 2007). However, the specific roles of reactive nitrogen species, such as nitric oxide (NO), in autophagy have been unclear. NO is a ubiquitous cellular messenger molecule in the cardiovascular, nervous, and immune systems, where NO is capable of eliciting a multitude of physiological responses, such as blood flow regulation and tissue responses to hypoxia (Foster et al., 2009). Endogenous NO is synthesized from L-arginine by a family of NO synthases (NOS) in a two-step oxidation process. NO-based protein modification by *S*-nitrosylation, which is the redox-based modification of Cys thiol side chains, is a common mechanism of NO-mediated signal transduction (Hess et al., 2005). Protein *S*-nitrosylation not only plays vital physiological roles that are important in cellular homeostasis, but also can also contribute to a broad spectrum of human diseases, such as heart failure and neurodegeneration (Foster et al., 2009). An excess of NO resulting in nitrosative stress is believed to play a causal role in neuronal cell death in the context of neurodegenerative diseases (Gu et al., 2010).

Here we show that NO inhibits autophagic flux in mammalian cells. NO *S*-nitrosylates JNK1 and IKKβ, and our data suggest that these events affect autophagy, as NO decreases JNK1 activity and Bcl-2 phosphorylation and activates mTORC1 in an IKKβ- and TSC2-dependent manner. Overexpression of NOS isoforms also impairs autophagic flux. Conversely, inhibition of NO synthesis induces autophagy and protects against neurodegeneration in models of Huntington's disease (HD). Our data delineate the role of NO in regulating autophagy, which may have underlying implications in its myriad of cellular functions.

## Results

### NO Inhibits Autophagosome Synthesis

**Figure 1:**
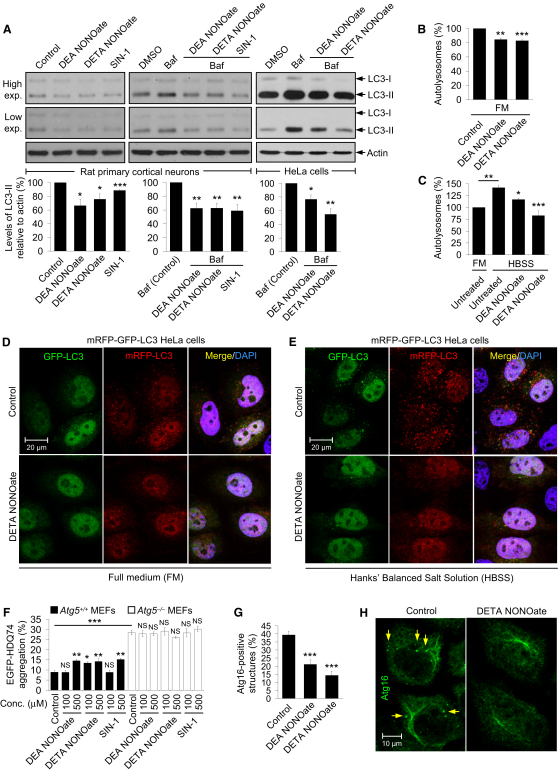
NO Inhibits Autophagosome Synthesis (A) Immunoblot analysis with anti-LC3 antibody shows that NO donors reduced LC3-II levels in rat primary cortical neurons and decreased autophagosome synthesis in bafilomycin A_1_-treated rat primary cortical neurons and HeLa cells. (B–E) Confocal microscopy images and analysis of autophagic flux by automated Cellomics microscopy show that NO donors reduced autolysosome numbers in mRFP-GFP-LC3 HeLa cells grown in full medium (FM) (B and D) or starved with HBSS (C and E). Cells in HBSS had more autolysosomes compared to FM (C). Autolysosome number in untreated cells in FM is set at 100%. (F) NO donors increased EGFP-HDQ74 aggregates in a dose-dependent manner in *EGFP-HDQ74*–transfected *Atg5^+/+^*, but not in *Atg5*^−/−^, MEFs. *Atg5*^−/−^ MEFs had more EGFP-HDQ74 aggregates than *Atg5^+/+^* MEFs. (G and H) Confocal microscopy images (H) and immunofluorescence analysis with anti-Atg16 antibody (G) show that NO donors reduced the percentage of HeLa cells with Atg16-positive structures (arrows) under starvation. Graphical data denote mean ± SEM.

We tested whether NO regulates autophagy using a number of NO-releasing chemical compounds (Keefer et al., 1996), such as DEA NONOate, DETA NONOate, and SIN-1. These NO donors decreased endogenous LC3-II levels in rat primary cortical neurons and HeLa cells, suggesting that NO may be inhibiting autophagy (Figure 1A; see Figure S1A available online). Because the steady state levels of LC3-II are affected both by synthesis and degradation, effects on autophagy are best assessed by clamping LC3-II/autophagosome degradation with bafilomycin A_1_ (see Experimental Procedures). This is a well-established method for monitoring autophagosome synthesis (Rubinsztein et al., 2009; Sarkar et al., 2007, 2009c). NO donors also reduced LC3-II levels in bafilomycin A_1_-treated rat primary cortical neurons or HeLa cells, compared to bafilomycin A_1_ treatment alone (control) (Figure 1A), indicating that NO impaired autophagosome synthesis.

We next analyzed autophagic flux in a stable HeLa cell line expressing mRFP-GFP-LC3 reporter, in which autophagosomes have both mRFP and GFP signals, whereas the autolysosomes emit only mRFP signal because of quenching of the GFP in the acidic lysosomal environment (Kimura et al., 2007; Sarkar et al., 2009a). NO donors decreased the number of autolysosomes in mRFP-GFP-LC3 HeLa cells cultured in full medium (FM) or when starved with Hank's buffered salt solution (HBSS), compared with untreated (control) cells (Figures 1B–1E), suggesting that NO inhibited autophagic flux under basal and starvation conditions.

To further verify our data on autophagic flux, we assessed the accumulation of EGFP-tagged mutant huntingtin (EGFP-HDQ74), a well-established autophagy substrate, where the proportion of cells with aggregates formed by this protein correlates linearly with expression levels (Ravikumar et al., 2004). NO donors increased the percentage of cells with EGFP-HDQ74 aggregates in *Atg5^+/+^* (autophagy-competent) MEFs, but not in *Atg5*^−/−^ (autophagy-deficient) MEFs, which is consistent with an effect on autophagy inhibition (Figure 1F and Figure S1B). Furthermore, NO donors decreased the number of endogenous starvation-induced Atg16-positive structures in HeLa cells (Figures 1G and 1H). Because Atg16 is located on the phagophores and is not found on the autophagosomes (Mizushima et al., 2003), these data suggest that NO impaired autophagosome formation at an early stage.

### NO Inhibits Autophagy Independently of the cGMP Pathway

A major signaling pathway mediating the effects of NO is the activation of guanylate cyclase leading to the formation of cyclic guanosine-3′5′-monophosphate (cGMP), which, in turn, activates cGMP-dependent protein kinase G (PKG) (Denninger and Marletta, 1999). To test whether cGMP regulates autophagy, we used 8-pCPT-cGMP, a cell-permeable analog of cGMP and a selective PKG activator (Geiger et al., 1992). HeLa cells treated with 8-pCPT-cGMP had no effects on LC3-II levels or autophagosome synthesis (Figure S1C). Likewise, 8-pCPT-cGMP had no effects on autolysosome numbers in mRFP-GFP-LC3 HeLa cells, or on the accumulation of EGFP-HDQ74 (Figures S1D and S1E), indicating that cGMP did not affect autophagy. Although 8-pCPT-cGMP had no effects on autophagic flux, DETA NONOate reduced LC3-II levels in the presence or absence of 8-pCPT-cGMP to a similar extent in bafilomycin A_1_–treated HeLa cells, suggesting that NO-mediated autophagy inhibition was independent of the cGMP pathway (Figure S1F).

### NO Increases Bcl-2–Beclin 1 Association by Inhibiting JNK1-Mediated Bcl-2 Phosphorylation

**Figure 2:**
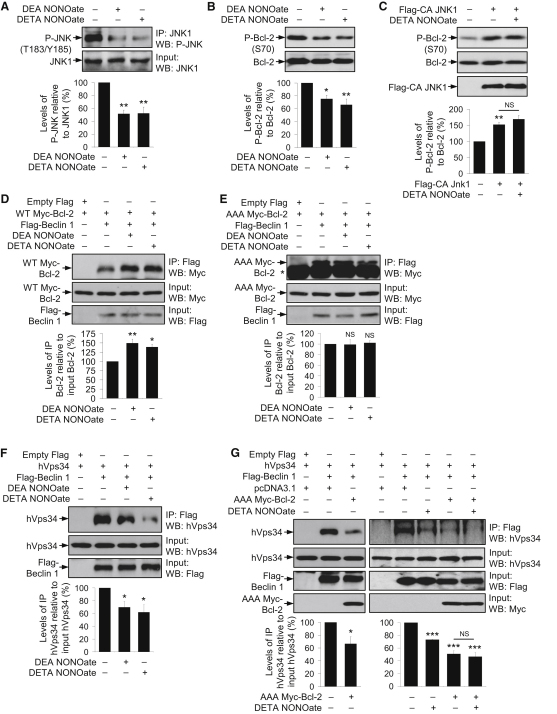
NO Reduced Phosphorylation of JNK1 and Bcl-2, Leading to Increased Bcl-2–Beclin 1 and Decreased Beclin 1–hVps34 Interactions (A) NO donors reduced JNK1 phosphorylation in HeLa cells, as analyzed by immunoprecipitation with anti-JNK1 agarose-conjugated beads and immunoblotting with anti-phospho-JNK antibody. (B) NO donors reduced Bcl-2 phosphorylation in HeLa cells, as analyzed by immunoblotting with anti-phospho-Bcl-2 antibody. (C) Immunoblot analysis with anti-phospho-Bcl-2 antibody shows that DETA NONOate could not reduce phospho-Bcl-2 in *Flag-CA JNK1*-transfected HeLa cells, where phospho-Bcl-2 was higher compared to mock-transfected cells. (D and E) Immunoprecipitation with anti-Flag M2 affinity agarose gel and immunoblotting with anti-Myc antibody shows that NO donors increased the interaction of Flag-Beclin 1 with WT Myc-Bcl-2 (D), but not with AAA Myc-Bcl-2 (E), in HeLa cells transfected with *WT Myc-Bcl-2* (D) or *AAA Myc-Bcl-2* (E) along with either *empty Flag* or *Flag-Beclin 1*. Asterisk denotes IgG band (E). (F) Immunoprecipitation with anti-Flag M2 affinity agarose gel and immunoblotting with anti-Vps34 antibody shows that NO donors decreased Flag-Beclin 1–hVps34 interaction in HeLa cells transfected with *hVps34* along with either *empty Flag* or *Flag-Beclin 1*. (G) Immunoprecipitation with anti-Flag M2 affinity agarose gel and immunoblotting with anti-Vps34 antibody shows that AAA Myc-Bcl-2 decreased Flag-Beclin 1–hVps34 interaction in the presence (right panel) or absence (left panel) of DETA NONOate in HeLa cells transfected with *hVps34* along with either *empty Flag* or *Flag-Beclin 1*, and with *pcDNA3.1* or *AAA Myc-Bcl-2*. Graphical data denote mean ± SEM.

Activation of JNK1, but not JNK2, has been recently shown to mediate starvation-induced autophagy in mammalian cells by phosphorylating Bcl-2 (Wei et al., 2008). NO donors can inhibit JNK1 via *S*-nitrosylation at C116, leading to reduced JNK1 phosphorylation (Hess et al., 2005; Park et al., 2000). Furthermore, JNK1 inhibition impairs both starvation-induced and basal autophagy (Wei et al., 2008). To test the possibility that NO-mediated autophagy inhibition may be acting via JNK1 inactivation, we confirmed that NO donors *S*-nitrosylated JNK1 and reduced endogenous JNK1 phosphorylation in HeLa cells (Figure 2A and Figure S2A). NO donors also decreased endogenous Bcl-2 phosphorylation in HeLa cells and human cortical neurons (Figure 2B and Figure S2B), but not in cells expressing constitutively active JNK1 (Figure 2C). These data suggest that NO reduced Bcl-2 phosphorylation by JNK1 inhibition.

Because starvation induces autophagy by increasing Bcl-2 phosphorylation and consequently disrupting the autophagy-inhibitory association of Bcl-2 and Beclin 1 (Pattingre et al., 2005), we next tested whether NO influences this interaction by reducing phospho-Bcl-2. HeLa cells expressing Flag-tagged Beclin 1 (Flag-Beclin 1) and Myc-tagged Bcl-2 (Myc-Bcl-2) showed increased binding of Bcl-2 to Beclin 1 upon treatment with NO donors, suggesting that the autophagy inhibitory effects of NO were due to increased association of the anti-autophagy protein Bcl-2 with Beclin 1 (Figure 2D). However, the effect of NO donors on Bcl-2–Beclin 1 interaction was abolished in cells expressing Flag-Beclin 1 and nonphosphorylatable Myc-Bcl-2 (AAA Myc-Bcl-2) with mutations at the T69A, S70A and S87A phosphorylation sites (Wei et al., 2008) (Figure 2E), suggesting that NO-mediated increased Bcl-2–Beclin 1 association was due to inhibition of JNK1-dependent Bcl-2 phosphorylation.

Bcl-2 can also be *S*-nitrosylated by NO in response to apoptotic stimuli at C158 and C229, with C229 being the major site as its mutation abrogated Bcl-2 *S*-nitrosylation (Azad et al., 2006). Using a mutant Bcl-2 construct that cannot be *S*-nitrosylated (C229A Bcl-2) (Azad et al., 2006), we found that NO donors not only increased Bcl-2–Beclin 1 association in Flag-Beclin 1 and Bcl-2 expressing cells, but also in Flag-Beclin 1 and C229A Bcl-2 expressing cells (Figures S2C and S2D). Therefore, the effect of NO in increasing Bcl-2–Beclin 1 interaction was independent of Bcl-2 *S*-nitrosylation on C229.

### NO Impairs the Formation of Beclin 1/hVps34 Autophagy Initiation Complex

Beclin 1 regulates the initiation of autophagosome formation as a part of the hVps34/PI3K complex (Pattingre et al., 2008). Consistent with NO-mediated increase in Bcl-2–Beclin 1 association, NO donors reduced Beclin 1–hVps34 interaction in cells expressing Flag-Beclin 1 and hVps34, suggesting that NO disrupted Beclin 1/hVps34 complex formation that is essential for autophagy initiation (Figure 2F). Furthermore, lowered Bcl-2 phosphorylation influenced Beclin 1–hVps34 association, because cells expressing Flag-Beclin 1 and hVps34 had reduced interaction in the presence of AAA Myc-Bcl-2, indicating that dephosphorylation of Bcl-2 is associated with an impairment of Beclin 1/hVps34 complex formation (Figure 2G). Moreover, AAA Myc-Bcl-2 reduced Beclin 1–hVps34 interaction to a similar extent in the presence or absence of DETA NONOate (Figure 2G). Collectively, these data implicate that NO-mediated Bcl-2 dephosphorylation sequestered Beclin 1 from the Beclin 1/hVps34 autophagy initiation complex into the Bcl-2/Beclin 1 autophagy inhibitory complex.

### NO Inhibits Autophagy by Additional Mechanisms Apart from the JNK1–Bcl-2–Beclin 1 Pathway

**Figure 3:**
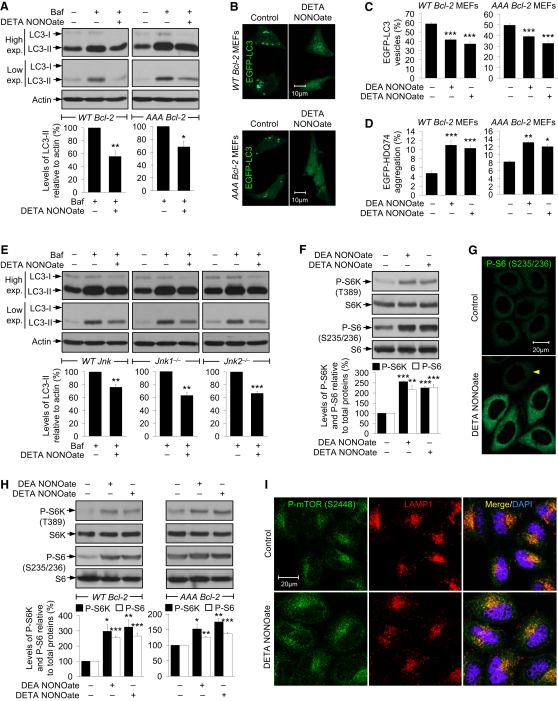
NO Impairs Autophagy in *AAA Bcl-2* and *Jnk1*^−/−^ MEFs by Activating mTORC1 (A) Immunoblot analysis with anti-LC3 antibody shows that DETA NONOate reduced autophagosome synthesis in bafilomycin A_1_-treated *WT Bcl-2* and *AAA Bcl-2* MEFs. (B and C) NO donors decreased EGFP-LC3 vesicles in *EGFP-LC3*–transfected *WT Bcl-2* and *AAA Bcl-2* MEFs (C). Images were acquired by a confocal microscope (B). *WT Bcl-2* and *AAA Bcl-2* MEFs were analyzed separately. (D) NO donors increased EGFP-HDQ74 aggregates in *EGFP-HDQ74*–transfected *WT Bcl-2* and *AAA Bcl-2* MEFs. *WT Bcl-2* and *AAA Bcl-2* MEFs were analyzed separately. (E) Immunoblot analysis with anti-LC3 antibody shows that DETA NONOate reduced autophagosome synthesis in bafilomycin A_1_-treated *WT Jnk*, *Jnk1*^−/−^, and *Jnk2*^−/−^ MEFs. (F) Immunoblot analyses with anti-phospho-S6K and anti-phospho-S6 antibodies show that NO donors activated mTORC1 in HeLa cells. (G) Confocal microscope images of immunofluorescence with anti-phospho-S6 antibody show that DETA NONOate increased S6 phosphorylation; arrowhead shows a cell where this effect was not observed. (H) Immunoblot analyses with anti-phospho-S6K and anti-phospho-S6 antibodies show that NO donors activated mTORC1 in *WT Bcl-2* and *AAA Bcl-2* MEFs. (I) Confocal microscope images of immunofluorescence with anti-phospho-mTOR and anti-LAMP1 antibodies in HeLa cells show that DETA NONOate increased phospho-mTOR but did not alter its distribution with lysosomes. Graphical data denote mean ± SEM.

In order to test whether NO-mediated autophagy inhibition was acting solely via the JNK1–Bcl-2–Beclin 1 pathway, we used MEFs stably expressing human wild-type Bcl-2 (*WT Bcl-2*) or AAA mutant Bcl-2 (*AAA Bcl-2*) (Wei et al., 2008). Interestingly, DETA NONOate inhibited autophagosome synthesis in both bafilomycin A_1_-treated *WT Bcl-2* and *AAA Bcl-2* MEFs (Figure 3A). NO donors also reduced EGFP-LC3 vesicles and increased EGFP-HDQ74 aggregation in both the cell lines (Figures 3B–3D).

We further studied the effects of NO in MEFs with targeted disruption of *jnk1* or *jnk2* genes (Tournier et al., 2000) (Figure S3A). Consistent with the effects of NO in *AAA Bcl-2* MEFs, DETA NONOate inhibited autophagosome synthesis in bafilomycin A_1_-treated *WT Jnk, Jnk1*^−/−^, or *Jnk2*^−/−^ MEFs (Figure 3E). These data indicate that NO impaired autophagy by additional mechanism(s) apart from modulating the JNK1–Bcl-2–Beclin 1 pathway.

### NO Activates mTORC1 to Negatively Regulate Autophagy

We next investigated the effects of NO on the classical mTOR pathway. The activity of mTORC1, a protein kinase, can be assessed by the phosphorylation status of its substrate, ribosomal S6 kinase 1 (S6K), at T389, and that of the ribosomal S6 protein (S6), a substrate of S6K, at S235/236 (Martin and Hall, 2005). NO donors increased the phosphorylation of S6K and S6 in HeLa cells, suggesting activation of mTORC1 (Figures 3F and 3G). Similar effects of NO were found in *WT Bcl-2* and *AAA Bcl-2* MEFs (Figure 3H). An elevated mTORC1 activity in *AAA Bcl-2* MEFs may account for NO inhibiting autophagy in these cells. DETA NONOate also activated mTORC1 in primary mouse and human cortical neurons, as well as in starved HeLa cells (Figures S3B and S3C), which are compatible with NO inhibiting neuronal and starvation-induced autophagy, respectively.

The regulation of mTORC1 activity by amino acids has been correlated with the localization of mTORC1 on late endosomes/lysosomes via the Rag GTPases (Sancak et al., 2008). Although DETA NONOate increased mTOR phosphorylation in HeLa cells, there was no overt redistribution of mTOR or phospho-mTOR, which primarily colocalized with LAMP1-positive late endosomal/lysosomal compartments near the perinuclear region similar to the untreated cells (Figure 3I and Figure S3D).

### Activation of mTORC1 by NO Is Dependent on TSC2 and IKKβ

**Figure 4:**
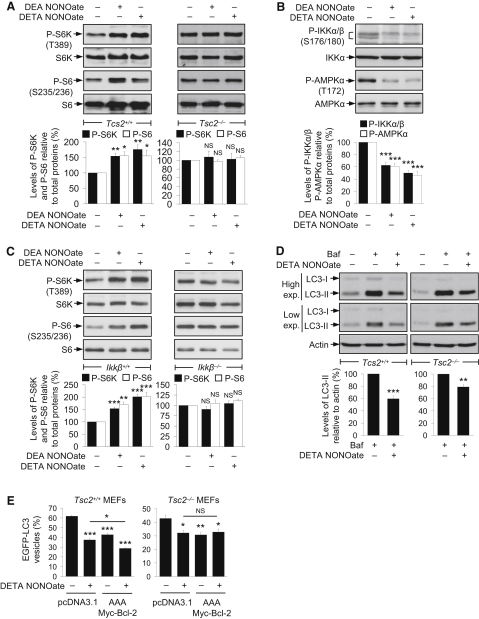
Activation of mTORC1 by NO Is Dependent on TSC2 and IKKβ (A) Immunoblot analyses with anti-phospho-S6K and anti-phospho-S6 antibodies show that NO donors activated mTORC1 in *Tsc2^+/+^* MEFs, but not in *Tsc2*^−/−^, MEFs. (B) NO donors reduced phosphorylation of IKKα/β and AMPKα in MEFs, as analyzed by immunoblotting with anti-phospho-IKKα/β and anti-phospho-AMPKα antibodies, respectively. (C) Immunoblot analyses with anti-phospho-S6K and anti-phospho-S6 antibodies show that NO donors activated mTORC1 in *Ikkβ^+/+^* MEFs, but not in *Ikkβ*^−/−^ MEFs. (D) Immunoblot analysis with anti-LC3 antibody shows that DETA NONOate reduced autophagosome synthesis in bafilomycin A_1_-treated *Tsc2^+/+^* and *Tsc2*^−/−^ MEFs. (E) DETA NONOate decreased EGFP-LC3 vesicles in *Tsc2^+/+^* and *Tsc2*^−/−^ MEFs transfected with *EGFP-LC3*, along with either *pcDNA3.1* or *AAA Myc-Bcl-2*. Although it further reduced EGFP-LC3 vesicles in *Tsc2^+/+^* MEFs expressing AAA Myc-Bcl-2 compared to mock-transfected *Tsc2^+/+^* MEFs, this effect was not seen in *Tsc2*^−/−^ MEFs. *Tsc2^+/+^* and *Tsc2*^−/−^ MEFs were analyzed separately. Graphical data denote mean ± SEM.

The tuberous sclerosis complex (TSC1/2), consisting of a heterodimer of TSC1 and TSC2, is an upstream negative regulator of mTORC1. TSC1/2 is the GTPase-activating protein for the Ras-family GTP-binding protein Rheb, which directly binds and activates mTORC1 (Guertin and Sabatini, 2009). Loss of TSC1 or TSC2 activity stimulates mTORC1 by enhancing Rheb activity (Figure S4A), which also impairs basal autophagy (Ravikumar et al., 2004; Williams et al., 2008; Zhou et al., 2009). Because the TSC1/2 complex regulates mTORC1 activity, we studied the effects of NO on mTORC1 in *Tsc2^+/+^* and *Tsc2*^−/−^ MEFs (Zhang et al., 2003). We found that NO donors increased the phosphorylation of S6K and S6 in *Tsc2^+/+^*, but not in *Tsc2*^−/−^, MEFs (Figure 4A), suggesting that NO-induced activation of mTORC1 was TSC2-dependent, by acting at, or above, TSC2.

NO *S*-nitrosylates and inhibits IKKβ. However, the active IKK complex normally regulates autophagy by enhancing AMPK phosphorylation (possibly indirectly), which, in turn, inhibits mTOR via TSC1/2 (Criollo et al., 2010; Lee et al., 2007; Reynaert et al., 2004). To test whether NO activates mTOR by this pathway, we confirmed that NO donors *S*-nitrosylated IKKβ and reduced its phosphorylation (Figure 4B and Figure S4B), leading to decreased AMPKα phosphorylation at the site phosphorylated by IKK (Figure 4B and Figure S4C). Because NO decreases AMPK phosphorylation, we predicted that NO may enhance mTORC1 activity by this pathway (Inoki et al., 2003). Indeed, NO donors increased mTORC1 activity in *Ikkβ^+/+^*, but not in *Ikkβ*^−/−^, MEFs (Figure 4C). We also found increased mTORC1 activity in *Ikkβ*^−/−^ MEFs, compared to *Ikkβ^+/+^* MEFs (Figure S4D). These data collectively indicate that NO-mediated mTORC1 activation is dependent on IKK and TSC2 activity.

### NO Inhibits Autophagy by Two Independent Pathways

We further tested the effects of NO on autophagy in *Tsc2^+/+^* and *Tsc2*^−/−^ MEFs, where DETA NONOate impaired autophagosome synthesis in both the cell lines treated with bafilomycin A_1_ (Figure 4D). Treatment with DETA NONOate or overexpression of AAA Myc-Bcl-2 also reduced EGFP-LC3 vesicle numbers in both *Tsc2^+/+^* and *Tsc2*^−/−^ MEFs (Figure 4E). However, DETA NONOate could further reduce EGFP-LC3 vesicles in AAA Myc-Bcl-2 expressing *Tsc2^+/+^* MEFs, but not in *Tsc2*^−/−^ MEFs, suggesting that the autophagy inhibition caused by NO was mediated predominantly (or maybe entirely) by the JNK1–Bcl-2 and mTORC1 pathways (Figure 4E).

Interestingly, AAA Myc-Bcl-2 expression decreased EGFP-LC3 vesicles even in *Tsc2*^−/−^ MEFs (Figure 4E). Moreover, we found no change in Bcl-2 phosphorylation in HeLa cells treated with or without the mTOR inhibitor rapamycin, or between *Tsc2^+/+^* and *Tsc2*^−/−^ MEFs (Figures S4E and S4F). Furthermore, overexpression of constitutively active JNK1 had no effects on mTOR activity (Figure S4G), suggesting that the Bcl-2–Beclin 1 and mTORC1 pathways are independent of each other. These data implicate that NO impairs autophagy by two distinct mechanisms via the JNK1–Bcl-2–Beclin 1 and IKK–AMPK–TSC2–mTORC1 pathways.

### Overexpression of NOS Isoforms Impairs Autophagosome Synthesis

Endogenous NO is generated by NOS, which has three mammalian isoforms arising from separate gene products: neuronal NOS (nNOS or NOS1), inducible NOS (iNOS or NOS2), and endothelial NOS (eNOS or NOS3). Although nNOS and eNOS are Ca^2+^-dependent enzymes producing NO, iNOS is a Ca^2+^-independent enzyme that produces NO when its expression is induced (Hess et al., 2005).

**Figure 5:**
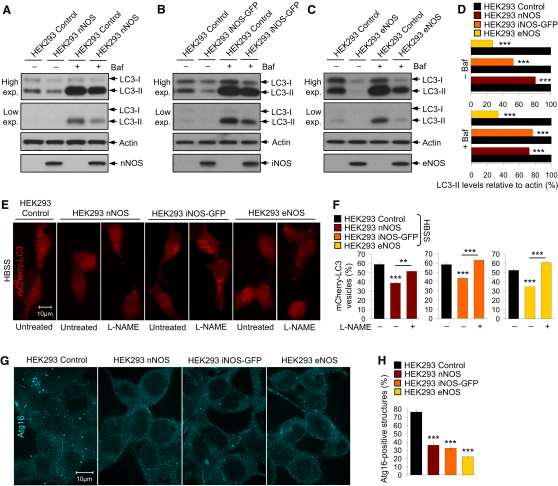
Overexpression of Mammalian NOS Isoforms Inhibits Autophagosome Synthesis (A–D) Immunoblot analyses with anti-LC3, along with anti-nNOS, anti-iNOS, and anti-eNOS antibodies, show decreased LC3-II levels in stable HEK293 cell lines overexpressing nNOS (A and D), iNOS-GFP (B and D), or eNOS (C and D) compared to HEK293 control cells in the presence or absence of bafilomycin A_1_. (E and F) mCherry-LC3 vesicles were reduced in *mCherry-LC3–*transfected stable HEK293 NOS cell lines compared to HEK293 control cells cultured in HBSS, an effect that was restored in the NOS cells by L-NAME (F). Images were acquired by a confocal microscope (E). (G and H) Confocal microscopy images (G) and immunofluorescence analysis with anti-Atg16 antibody show that stable HEK293 NOS cell lines had fewer Atg16-positive structures under starvation, compared to HEK293 control cells (H). Graphical data denote mean ± SEM.

To test whether NOS overexpression recapitulated the effects of NO donors on autophagy, we used stable HEK293 cell lines expressing nNOS, iNOS-GFP, or eNOS (Figures S5A–S5E). Because of the absence of endogenous NOS isoforms in HEK293 (control) cells, the stable HEK293 NOS cell lines have elevated basal NO levels compared to the control cells (Kornberg et al., 2010; Liu et al., 1996; Pandit et al., 2009; Schmidt et al., 2001). We found that LC3-II levels and autophagosome synthesis were markedly reduced in all the NOS-expressing cell lines grown in the absence or presence of bafilomycin A_1_, compared to the control cells (Figures 5A–5D). Likewise, the NOS-expressing cells had reduced LC3-II levels and mCherry-LC3 vesicle numbers under starvation conditions, compared to the control cells; this effect was partly or wholly rescued by a broad-spectrum NOS inhibitor, *N_ω_*-nitro-L-arginine methyl ester hydrochloride (L-NAME) (Figures 5E and 5F and Figures S5F–S5J). Furthermore, the number of endogenous starvation-induced Atg16-positive structures was lower in all the NOS-expressing cells, compared to the control cells (Figures 5G and 5H). Therefore, overexpression of the NOS isoforms impairs basal and starvation-induced autophagy at an early stage of autophagosome synthesis.

### NOS Overexpression Inhibits the JNK1–Bcl-2 Pathway

**Figure 6:**
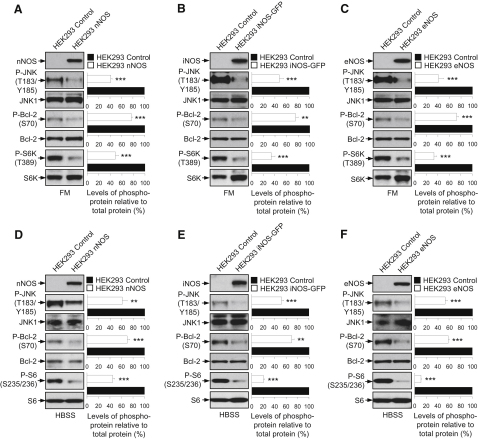
NOS Overexpression Primarily Inhibits the JNK1–Bcl-2 Pathway to Impair Autophagy (A–F) Immunoblot analyses with anti-nNOS, anti-iNOS, anti-eNOS, anti-phospho-JNK1, anti-phospho-Bcl-2, anti-phospho-S6K, and anti-phospho-S6 antibodies show that stable HEK293 cell lines overexpressing nNOS (A and D), iNOS-GFP (B and E), or eNOS (C and F) inhibited both the JNK1–Bcl-2 and mTORC1 pathways compared to the HEK293 control cells under basal (FM) (A–C) or starvation (HBSS) (D–F) conditions. Graphical data denote mean ± SEM.

We next tested the NO signaling pathways regulating autophagy in the HEK293 NOS stable cells under basal (FM) and starvation (HBSS) conditions. We found that the phosphorylation of JNK1 and Bcl-2 were reduced in all the NOS-expressing cells compared to the control cells cultured in FM or HBSS (Figures 6A–6F), compatible with the effects of NO donors. Surprisingly, the phosphorylation of S6K in FM or S6 in HBSS was also decreased in the NOS stable cells, which is in contrast to the NO donors that activated mTORC1 (Figures 6A–6F). (Both phospho-S6K and phospho-S6 levels are reduced by starvation, although the levels of the latter are still sufficient to allow detection of a further decrease.) We speculate that these NOS cell lines may be chronically starved as a result of decreased autophagy, and this possibly inhibits the mTOR. Indeed, if one blocks autophagy for a prolonged period with a range of different chemical inhibitors, one also sees an eventual decrease in mTORC1 activity (Figure S6). Thus, autophagy is impaired in the NOS-expressing cells predominantly by inhibition of the JNK1–Bcl-2 pathway.

### NOS Inhibitor L-NAME Induces Autophagy Independently of the Pathways Perturbed by NO

**Figure 7:**
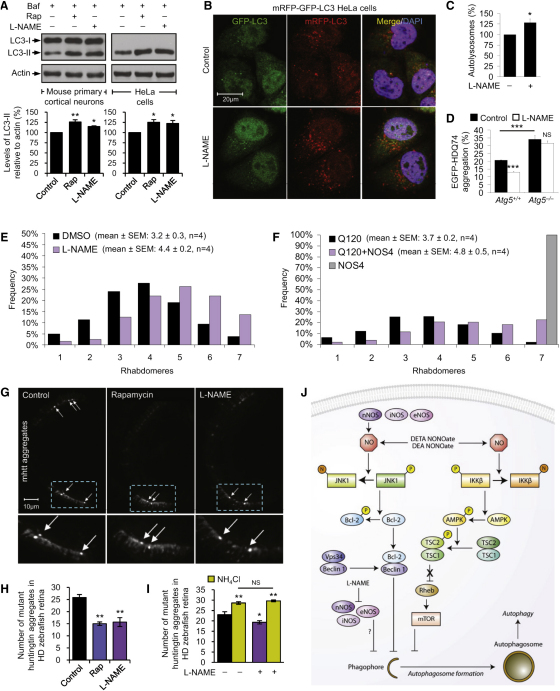
L-NAME Induces Autophagy and Reduces Mutant Huntingtin Aggregation/Neurodegeneration in Models of Huntington's Disease (A) Immunoblot analyses with anti-LC3 antibody show that rapamycin (8 hr) and L-NAME (24 hr) increased autophagosome synthesis in bafilomycin A_1_-treated mouse primary cortical neurons and HeLa cells. (B and C) Confocal microscopy images (B) and analysis of autophagic flux by automated Cellomics microscope (C) show that L-NAME increased autolysosomes in mRFP-GFP-LC3 HeLa cells. (D) L-NAME reduced EGFP-HDQ74 aggregates in *EGFP-HDQ74*–transfected *Atg5^+/+^*, but not in *Atg5*^−/−^, MEFs. (E) *Drosophila* expressing mutant huntingtin exon 1 (Q120) shows a significant decrease in neurodegeneration (p < 0.001, paired t test) upon L-NAME treatment compared to DMSO. (F) Expression of an endogenous negative regulator of NOS activity (NOS4) significantly attenuates neurodegeneration (p < 0.05, paired t test) in *Drosophila* expressing mutant huntingtin exon 1 (Q120). (G–I) Images from the retina of transgenic HD zebrafish show mutant huntingtin aggregates (arrows) (G). Treatment with rapamycin or L-NAME reduced the number of aggregates (H). L-NAME did not reduce aggregates in the presence of NH_4_Cl, which increased aggregate count (I). (J) NO inhibits autophagy by *S*-nitrosylating and inhibiting JNK1 phosphorylation, thereby reducing phospho-Bcl-2 and increasing Bcl-2–Beclin 1 interaction, which disrupts hVps34–Beclin 1 association. NO also *S*-nitrosylates and inhibits IKKβ phosphorylation, leading to reduced phospho-AMPK and TSC2 activity, which alleviates the inhibitory effect of TSC1/2 on Rheb (denoted by “×”), thereby allowing Rheb to activate mTORC1 and inhibit autophagy. Overexpression of NOS isoforms impairs autophagy by inhibiting the JNK1 pathway, whereas NOS inhibition by L-NAME induces autophagy by mechanism distinct from the NO pathways. Graphical data denote mean ± SEM, except in (E) and (F).

We next investigated whether NOS inhibition induces autophagy using the broad-spectrum NOS inhibitor, L-NAME. Consistent with the effects of NO donors on autophagy inhibition, L-NAME increased autophagosome synthesis (comparable to rapamycin) in bafilomycin A_1_-treated mouse primary cortical neurons and HeLa cells under basal conditions (Figure 7A and Figure S7A). It also had similar effects in rat primary cortical neurons under basal conditions and in HeLa cells under starvation conditions (Figures S7A and S7B). L-NAME also increased autophagic flux quantified as the number of autolysosomes using the mRFP-GFP-LC3 reporter (Figures 7B and 7C). The induction of autophagy by L-NAME was due to NOS inhibition because it had no effect on autophagy in HEK293 cells (lacking NOS isoforms) under basal or starvation condition (Figures S7C–S7E).

In contrast to the autophagy signaling pathways perturbed by NO donors, induction of autophagy by L-NAME was independent of mTORC1 activity or Bcl-2 phosphorylation, because HeLa cells treated with L-NAME exhibiting elevated LC3-II levels had no change in the phosphorylation of S6K, S6, or Bcl-2 (Figure S7F). Moreover, it had no effects on Beclin 1 or the lysosomal hydrolase, cathepsin D (Figure S7F).

### NOS Inhibition Reduces Mutant Huntingtin Aggregation and Neurodegeneration in Models of Huntington's Disease

Autophagy is a critical pathway regulating the turnover of mutant huntingtin associated with HD (Ravikumar et al., 2004). Consistent with an effect on autophagy induction, L-NAME reduced the percentage of cells with EGFP-HDQ74 aggregates in autophagy-competent *Atg5^+/+^* MEFs, but not in autophagy-deficient *Atg5*^−/−^ MEFs (Figure 7D). Likewise, siRNA knockdown of nNOS also reduced the percentage of SK-N-SH human neuroblastoma cells with EGFP-HDQ74 aggregates (Figure S7G). L-NAME also enhanced the clearance of autophagy substrates (comparable to rapamycin) like EGFP-HDQ74 and A53T α-synuclein in stable inducible PC12 cell lines (Sarkar et al., 2009c) (Figures S7H and S7I).

Because autophagy inducers have been shown to be protective in various animal models of HD and neurodegenerative disorders (Rubinsztein et al., 2007; Sarkar et al., 2009b), we tested the efficacy of L-NAME in a *Drosophila* model of HD. Flies expressing a mutant huntingtin fragment with 120 glutamines (Q120) in their photoreceptors exhibit retinal degeneration, a phenomenon that is not observed in flies expressing the wild-type protein with 23 glutamines (Q23) (Jackson et al., 1998). The photoreceptor degeneration observed in Q120 flies was significantly attenuated after treatment with L-NAME (Figure 7E). Moreover, the *NOS* gene in *Drosophila* is subjected to a complex transcriptional and posttranscriptional regulation (Stasiv et al., 2001). The product of one of its numerous transcripts, NOS4, acts as an endogenous dominant-negative regulator of NOS activity (Stasiv et al., 2004). Using this genetic approach to inhibit NOS activity, we showed that NOS4 overexpression in the HD fly eye also significantly ameliorated Q120 toxicity (Figure 7F).

We further tested the therapeutic effects of L-NAME in a transgenic EGFP-HDQ71 zebrafish model of HD [Tg(rho:EGFP-HTT71Q)^cu5^] by assessing mutant huntingtin aggregates in the retina where the transgene is driven by the rhodopsin promoter (Williams et al., 2008). We found that L-NAME, as well as the autophagy inducer rapamycin, reduced the number of aggregates compared to the control, whereas autophagy inhibitors such as wortmannin and ammonium chloride (NH_4_Cl) increased aggregate count (Figures 7G and 7H and Figure S7J). The ability of L-NAME to reduce aggregates in HD zebrafish was abolished in the presence of NH_4_Cl, suggesting that its effect in vivo is autophagy dependent (Figure 7I).

## Discussion

### Multiple Mechanisms Regulating NO-Mediated Autophagy Inhibition

Our study identifies a role of NO in the regulation of autophagy (Figure 7J). NO impaired autophagy at an early stage of autophagosome formation. The effects of NO in decreasing autophagic flux were robust in various cell lines and were independent of the cGMP pathway, which is an important mediator of NO effects. Our data suggest that NO inhibits autophagy by two distinct mechanisms (Figure 7J). First, NO *S*-nitrosylates and inactivates JNK1, thereby reducing Bcl-2 phosphorylation, which increases Bcl-2–Beclin 1 interaction and disrupts Beclin 1–hVps34 association. Second, NO activates mTORC1 in a manner dependent on TSC2 and IKKβ and is associated with decreased AMPK phosphorylation by IKK, consistent with the previously described inhibitory effects of NO on IKKβ activity (Reynaert et al., 2004). Although NO inhibited autophagy in *Jnk1*^−/−^, *AAA Bcl-2*, and *Tsc2*^−/−^ MEFs, its effects were abrogated in *Tsc2*^−/−^ MEFs expressing AAA Bcl-2. Thus, NO negatively regulates autophagy via the JNK1–Bcl-2–Beclin1 and the IKK–AMPK–TSC2–mTOR pathways. These two pathways implicated in starvation-induced autophagy appear to be independent of each other, because modulation of mTORC1 activity had no effects on Bcl-2 phosphorylation, and constitutively active JNK1 had no effects on mTORC1 activity.

Our data suggest that different ROS have divergent effects on autophagy. Although superoxides induced autophagy (Chen et al., 2009), NO impaired autophagosome synthesis. Overexpression of nNOS, iNOS, or eNOS also inhibited autophagic flux primarily via the JNK1–Bcl-2 pathway. Interestingly, the induction of autophagy by NOS inhibition was independent of the signaling pathways perturbed by NO or NOS overexpression, suggesting that there may be additional pathways regulating autophagy that are sensitive to changes in NO levels. Another possibility may be that NOS directly affects the autophagy machinery downstream of these signaling pathways.

A recent study reported that the plant extract evodiamine, which elicits apoptosis and autophagy, can also induce NO (Yang et al., 2008). There may be no contradiction between these findings and our observations, because it is possible that the effects of evodiamine on autophagy were independent of NO, as this compound influences many other cellular processes and signaling cascades.

### Consequences of NO Modulation in Neurodegenerative Diseases

Pharmacological or genetic inhibition of NOS increased the clearance of mutant huntingtin, which is an established autophagy substrate associated with HD (Ravikumar et al., 2004). Treatment with L-NAME, or expression of NOS4 in flies that acts as an endogenous dominant negative regulator of NOS activity (Stasiv et al., 2004), protected against neurodegeneration in a fly model of HD. Likewise, L-NAME reduced mutant huntingtin aggregates in a HD zebrafish model but was ineffective in the presence of the autophagy inhibitor NH_4_Cl. This is consistent with the protective effects seen with several autophagy inducers in various models of neurodegenerative diseases (Rubinsztein et al., 2007; Sarkar et al., 2009b).

Decreasing NO levels may protect against mutant huntingtin fragment toxicity via multiple mechanisms. For instance, excitotoxicity resulting from overstimulation of NMDA receptors is a prominent mechanism that has been implicated in neurodegenerative conditions such as Alzheimer disease, Parkinson's disease, and HD. Excessive NO production has been proposed as one of the mediators of excitotoxicity (Gu et al., 2010). Although this has previously been suggested to enhance protein misfolding by inactivation of protein disulphide isomerase, HSP90, and related mechanisms, our data suggest that the ensuing inhibition of autophagy will also contribute to this phenomenon because this will increase the levels of the aggregate-prone autophagy substrates. Various intracytosolic, mutant proteins associated with neurodegenerative diseases are primarily degraded by autophagy, and an impairment of autophagy in such conditions may augment their toxicity (Ravikumar et al., 2005; Rubinsztein et al., 2007; Sarkar et al., 2009b). Inactivation of autophagy will also enhance the susceptibility of such neurons to apoptotic insults (Boya et al., 2005; Ravikumar et al., 2006). However, our data provide an additional way whereby NO inhibition may mediate beneficial effects in neurodegenerative diseases where there is evidence of nitrosative stress-induced cell death linked to the pathogenesis of such diseases (Foster et al., 2009).

## Experimental Procedures

Complete methods can be found in Supplemental Experimental Procedures.

### Immunoblot Analysis

Cell lysates were subjected to western blot, as previously described (Sarkar et al., 2009c).

### Autophagy Analysis

#### Analysis of Autophagosome Synthesis by LC3-II Levels

Autophagosome synthesis was analyzed by measuring LC3-II (immunoblotting with anti-LC3 antibody) in the presence of bafilomycin A_1_, as previously described (Rubinsztein et al., 2009; Sarkar et al., 2009c).

#### Analysis of Autophagic Flux by mRFP-GFP-LC3 with an Automated Cellomics Microscope

Autolysosomes were quantified with an automated Cellomics microscope, as previously described (Sarkar et al., 2009a).

#### Analysis of Autophagosome Numbers by EGFP-LC3 or mCherry-LC3 Vesicles

The percentage of EGFP- or mCherry-positive cells with >5 EGFP-LC3 or mCherry-LC3 vesicles was assessed with a fluorescence microscope, as previously described (Sarkar et al., 2007).

### Statistical Analyses

Statistical analyses were performed using STATVIEW v4.53 and SPSS 9, as previously described (Sarkar et al., 2009c). Statistical data in figures are detailed in Supplemental Notes (^∗∗∗^, p < 0.001; ^∗∗^, p < 0.01; ^∗^, p < 0.05; NS, nonsignificant).

## Supplemental Information

Document S1. Seven Figures, Supplemental Experimental Procedures, Supplemental References, and Supplemental Notes
