# Supplementary material for: Complex Inhibitory Effects of Nitric Oxide on Autophagy
Source: Mol Cell. 2011 Jul 8;43(1):19–32. doi: 10.1016/j.molcel.2011.04.029 (PMC3149661; doi:10.1016/j.molcel.2011.04.029)
Supplement: Document S1. Seven Figures, Supplemental Experimental Procedures, Supplemental References, and Supplemental Notes [file mmc1.pdf]

# Supplemental Information

Molecular Cell, *Volume 43*

## Complex Inhibitory Effects of Nitric Oxide on Autophagy

Sovan Sarkar, Viktor I. Korolchuk, Maurizio Renna, Sara Imarisio, Angeleen Fleming, Andrea Williams, Moises Garcia-Arencibia, Claudia Rose, Shouqing Luo, Benjamin R. Underwood, Guido Kroemer, Cahir J. O'Kane, and David C. Rubinsztein

## FIGURE S1: Relates to Figure 1

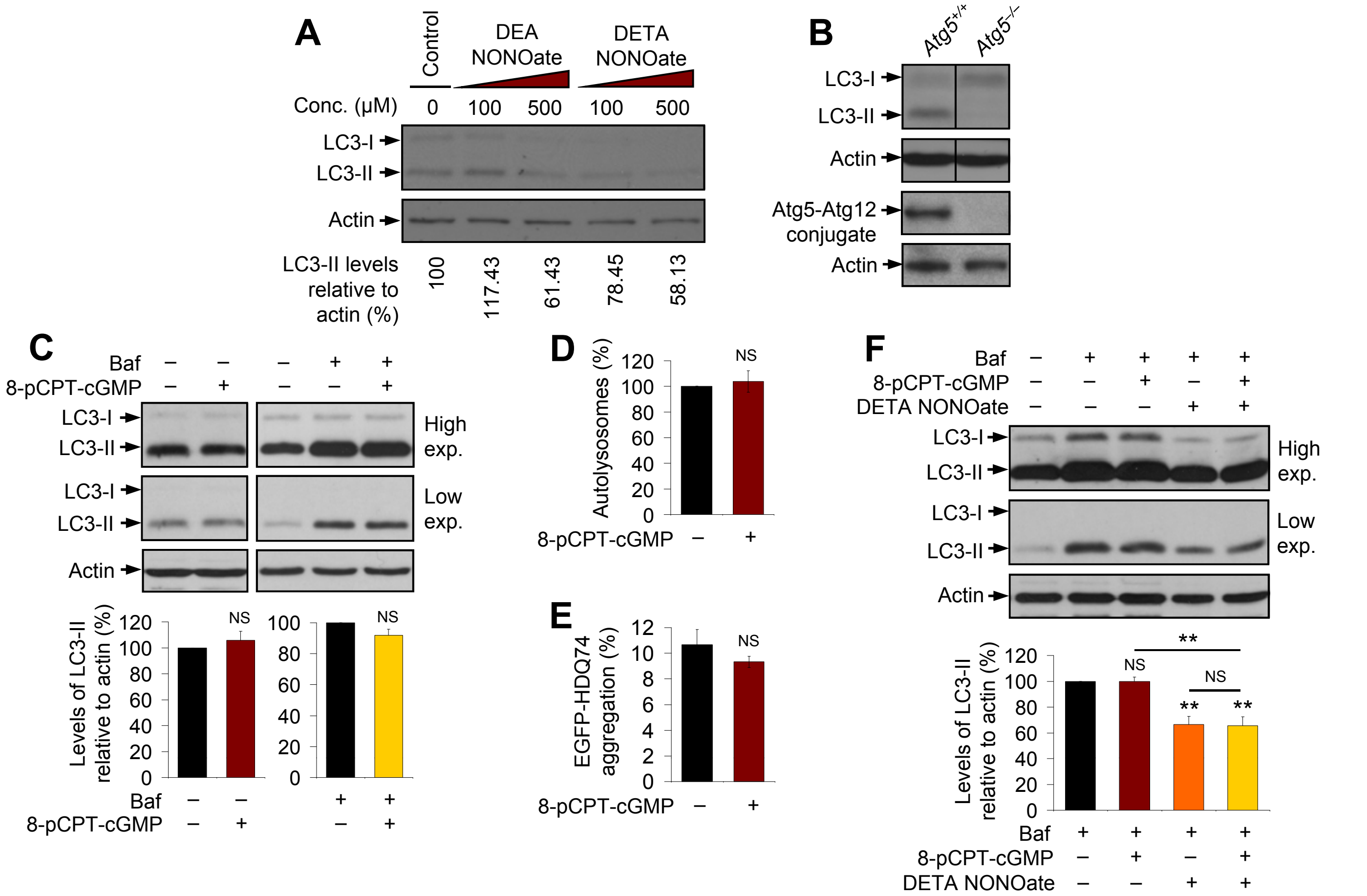


**Figure S1. Inhibition of Autophagy by NO Is Independent of the cGMP Pathway**

**(A)** Immunoblot analysis with anti-LC3 antibody shows that DEA NONOate and DETA NONOate reduced LC3-II levels in HeLa cells in a dose-dependent manner.

**(B)** Immunoblot analysis with anti-LC3 and anti-Atg5 antibodies show the absence of LC3-II and Atg5-Atg12 conjugate in *Atg5*$^{-/-}$ MEFs compared to *Atg5*$^{+/+}$ MEFs. Note that the lanes appearing as separate gel strips in the top panels were derived from non-adjacent lanes from the same exposure of immunoblot from a single gel where all the samples were loaded; the intervening lanes were excised to simplify presentation.

**(C)** Immunoblot analysis with anti-LC3 antibody shows that HeLa cells treated with 8-pCPT-cGMP had no significant changes in LC3-II levels or on autophagosome synthesis compared to the untreated cells, cultured in the absence ($p$=0.4427) or presence ($p$=0.1055) of bafilomycin $A_1$, suggesting that cGMP has no effects on autophagy.

**(D)** Analysis of autophagic flux by an automated Cellomics microscope shows that mRFP-GFP-LC3 HeLa cells treated with 8-pCPT-cGMP had no significant change in the number of autolysosomes compared to the untreated cells ($p$=0.6716).

**(E)** Assessment of *EGFP-HDQ74*–transfected HeLa cells with a fluorescence microscope shows that 8-pCPT-cGMP had no significant change in the proportion cells with EGFP-HDQ74 aggregates compared to the untreated cells ($p$=0.442), suggesting that 8-pCPT-cGMP has no effect on the clearance of autophagy substrate.

**(F)** Immunoblot analysis with anti-LC3 antibody shows that the effect of DETA NONOate in reducing autophagosome synthesis in bafilomycin $A_1$-treated HeLa cells ($p$=0.0013, without 8-pCPT-cGMP; $p$=0.0011, with 8-pCPT-cGMP) was not perturbed by the addition of 8-pCPT-cGMP, which itself had no effect on autophagosome synthesis ($p$=0.9952). As there was no significant change in LC3-II levels between bafilomycin $A_1$-treated cells cultured in the presence of both the compounds or DETA NONOate alone ($p$=0.8911), this suggests that inhibition of autophagic flux by nitric oxide is independent of the cGMP pathway.

Graphical data are represented as mean +/– SEM. **, $p$<0.01; NS, Non-significant.

# FIGURE S2: Relates to Figure 2

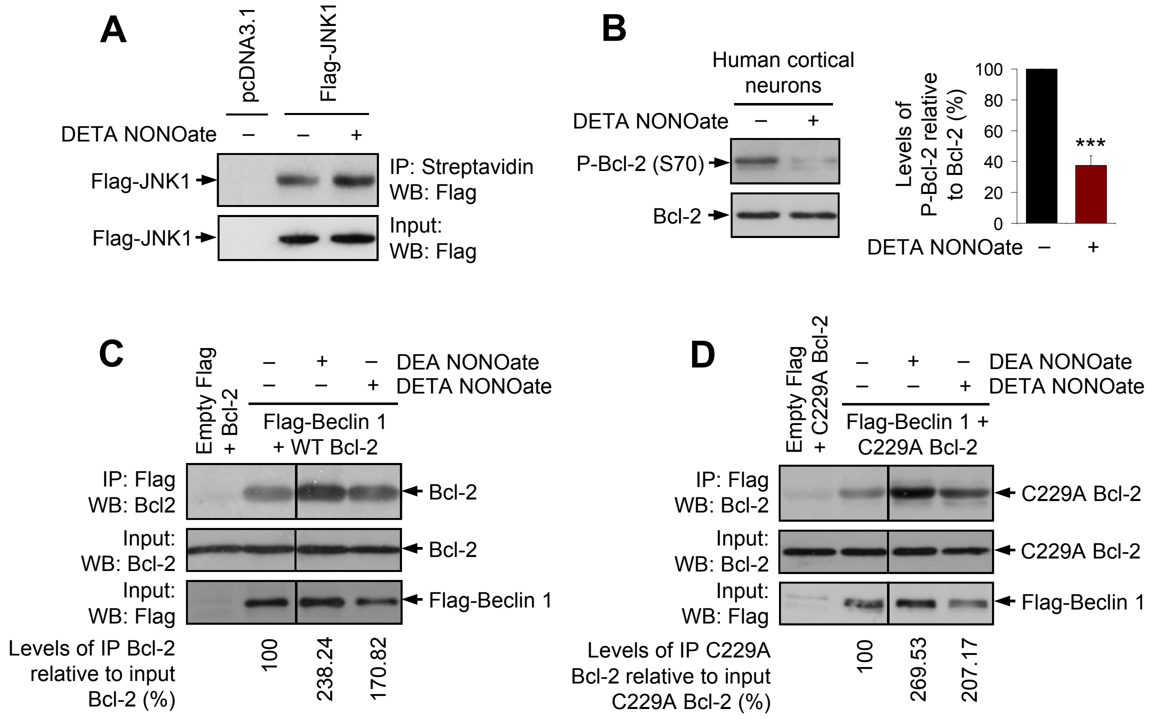


**Figure S2. NO *S*-Nitrosylates JNK1, Reduces Bcl-2 Phosphorylation, and Also Increases Bcl-2-Beclin 1 Interaction Independently of Bcl-2 *S*-Nitrosylation**

**(A)** *Flag-JNK1* or *pcDNA3.1* (empty vector)-transfected HeLa cells were subjected to biotin derivatization as described in Supplemental Experimental Procedures. Biotinylation of Flag-JNK1 was detected after precipitation with streptavidin agarose and immunoblotting using anti-Flag antibody. Fractions of samples used for precipitation with streptavidin agarose were also run on the immunoblot as inputs. Nitrosylation of Flag-JNK1 was increased in cells treated with DETA NONOate.

**(B)** Immunoblot analysis with anti-phospho-Bcl-2 (Ser70) antibody shows that DETA NONOate significantly reduced the phosphorylation of Bcl-2 in human cortical neurons ($p$<0.0001).

**(C)** Immunoprecipitation with anti-Flag M2 affinity agarose gel and immunoblotting with anti-Bcl-2 antibody shows that DEA NONOate and DETA NONOate increased the interaction of Flag-Beclin 1 with wild-type (WT) Bcl-2 in HeLa cells transfected with *WT Bcl-2* along with either *empty Flag* or *Flag-Beclin 1*. Note that the lanes appearing as separate gel strips were derived from non-adjacent lanes from the same exposure of immunoblot from a single gel where all the samples were loaded; the intervening lanes were excised to simplify presentation.

**(D)** Immunoprecipitation with anti-Flag M2 affinity agarose gel and immunoblotting with anti-Bcl-2 antibody shows that DEA NONOate and DETA NONOate increased the interaction of Flag-Beclin 1 with C229A Bcl-2 in HeLa cells transfected with *C229A Bcl-2* along with either *empty Flag* or *Flag-Beclin 1*, suggesting that the increase in Bcl-2–Beclin 1 association by nitric oxide is independent of Bcl-2 C229A *S*-nitrosylation. Note that the lanes appearing as separate gel strips were derived from non-adjacent lanes from the same exposure of immunoblot from a single gel where all the samples were loaded; the intervening lanes were excised to simplify presentation.

Graphical data is represented as mean +/– SEM. ***, $p$<0.001.

# FIGURE S3: Relates to Figure 3

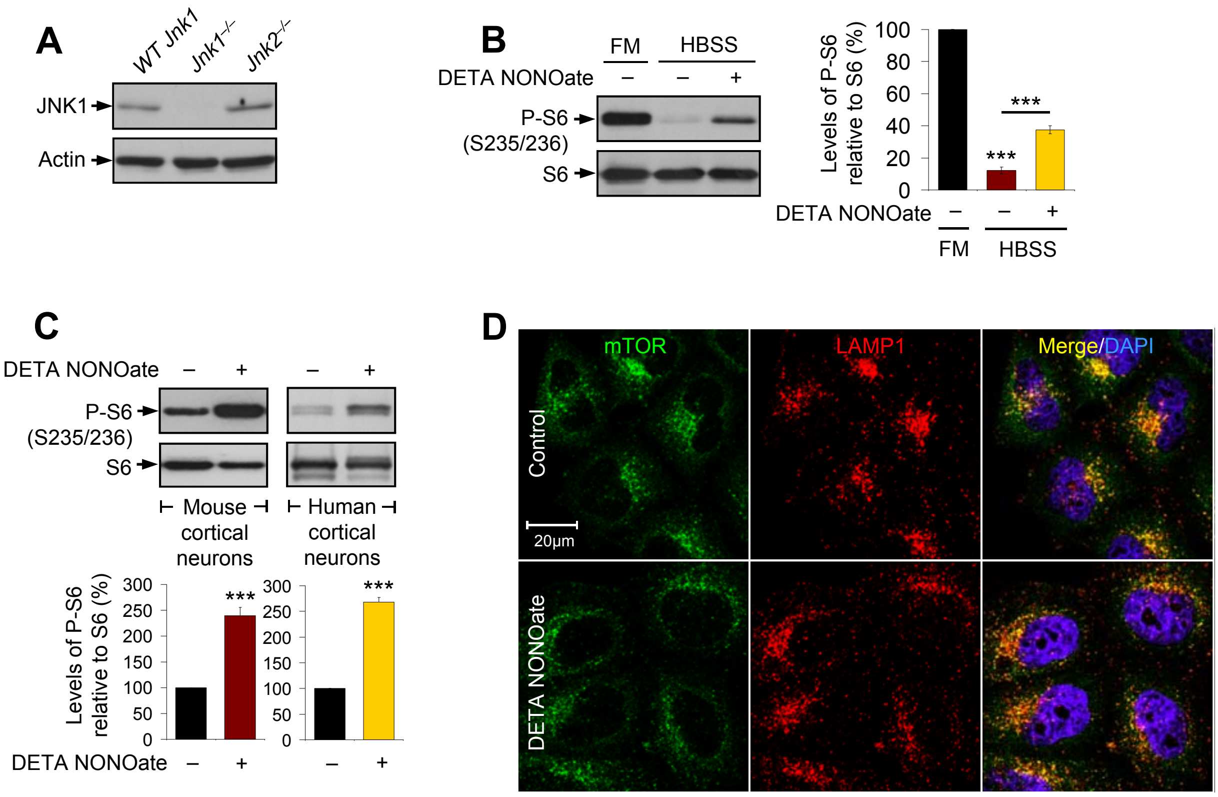


**Figure S3. NO Activates mTOR under Basal and Starvation Conditions**

**(A)** Immunoblot analysis with anti-JNK1 antibody in *WT Jnk1*, *Jnk1*$^{-/-}$ and *Jnk2*$^{-/-}$ MEFs confirms the absence of JNK1 in *Jnk1*$^{-/-}$ MEFs.

**(B)** Immunoblot analysis with anti-phospho-S6 (Ser235/236) antibody shows that DETA NONOate significantly elevated the phosphorylation of S6 in HeLa cells starved with Hank's Buffered Salt Solution (HBSS) ($p$<0.0001), suggesting that NO activates mTORC1 under starvation condition. Starved cells had significantly lower phospho-S6 levels compared to the cells cultured in full medium (FM) ($p$<0.0001).

**(C)** Immunoblot analysis with anti-phospho-S6 (Ser235/236) antibody shows that DETA NONOate significantly increased the phosphorylation of S6 in primary mouse ($p$<0.0001) and human cortical neurons ($p$<0.0001), suggesting that NO activates mTORC1 in neurons.

**(D)** Confocal microscope images of immunofluorescence with anti-mTOR and anti-LAMP1 antibodies in HeLa cells show that DETA NONOate did not alter the distribution of mTOR and lysosomes. Scale bar, 20 μm.

Graphical data are represented as mean +/– SEM. ***, $p$<0.001; **, $p$<0.01; *, $p$<0.05; NS, Non-significant.

## FIGURE S4: Relates to Figure 4

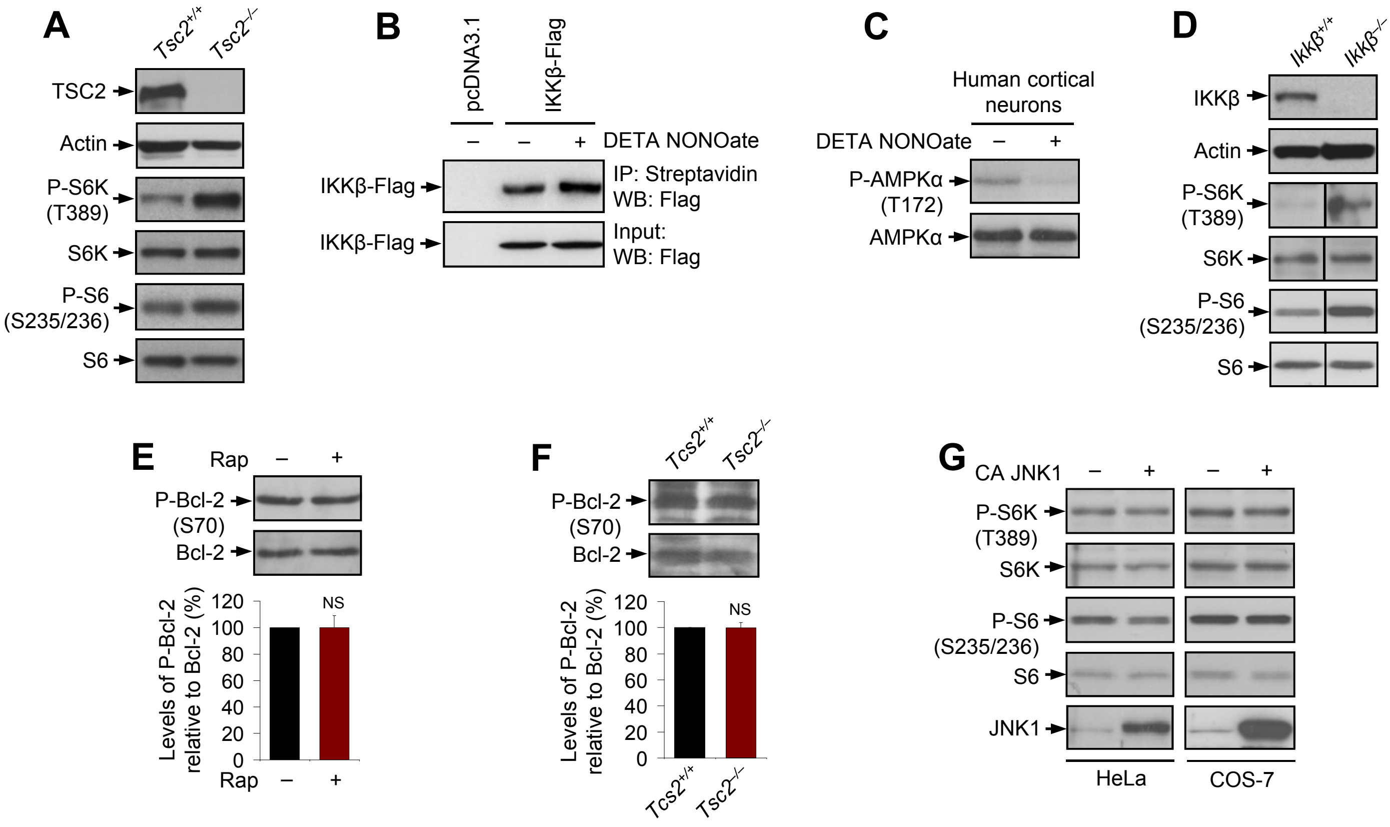


**Figure S4. NO *S*-Nitrosylates IKKβ and Reduces AMPK Phosphorylation, and the mTORC1 and JNK1–Bcl-2 Pathways Are Independent of Each Other**

**(A)** Immunoblot analyses with anti-TSC2, anti-phospho-S6K (Thr389) and anti-phospho-S6 (Ser235/236) antibodies confirm the absence of TSC2 and increased mTOR activity in *Tsc2*$^{-/-}$ MEFs compared to the *Tsc2*$^{+/+}$ MEFs.

**(B)** *IKKβ-Flag* or *pcDNA3.1* (empty vector)-transfected HeLa cells were subjected to biotin derivatization. Biotinylation of IKKβ-Flag was detected after precipitation with streptavidin-agarose and immunoblotting using anti-Flag antibody. Fractions of samples used for precipitation with streptavidin-agarose were also run on the immunoblot as inputs. Nitrosylation of IKKβ-Flag was increased in cells treated with DETA NONOate.

**(C)** Immunoblot analysis with anti-phospho-AMPKα (Ser176/180) antibody shows that DETA NONOate reduced the phosphorylation of AMPKα in human cortical neurons.

**(D)** Immunoblot analyses with anti-IKKβ, anti-phospho-S6K (Thr389) and anti-phospho-S6 (Ser235/236) antibodies confirm the absence of IKKβ and increased mTOR activity in *IKKβ*$^{-/-}$ MEFs compared to the *IKKβ*$^{+/+}$ MEFs. Note that the lanes appearing as separate gel strips in the lower panels were derived from non-adjacent lanes from the same exposure of immunoblot from a single gel where all the samples were loaded; the intervening lanes were excised to simplify presentation.

**(E)** Immunoblot analysis with anti-phospho-Bcl-2 (Ser70) antibody shows that there was no significant change in Bcl-2 phosphorylation in HeLa cells treated with rapamycin compared to the DMSO-treated cells ($p$=0.9906).

**(F)** Immunoblot analysis with anti-phospho-Bcl-2 (Ser70) antibody shows that there was no significant change in Bcl-2 phosphorylation in *Tsc2*$^{-/-}$ MEFs compared to the *Tsc2*$^{+/+}$ MEFs ($p$=0.9497).

**(G)** Immunoblot analyses with anti-phospho-S6K (Thr389), anti-phospho-S6 (Ser235/236) and anti-JNK1 antibodies in HeLa or COS-7 cells transfected with either *pcDNA3.1* (empty vector) or constitutively active (*CA) JNK1* show that there was no difference in mTOR activity in both the cell lines expressing constitutively active (CA) JNK1 compared to the empty vector transfected cells.

Graphical data are represented as mean +/– SEM. ***, $p$<0.001; **, $p$<0.01; NS, Non-significant.

# FIGURE S5: Relates to Figure 5

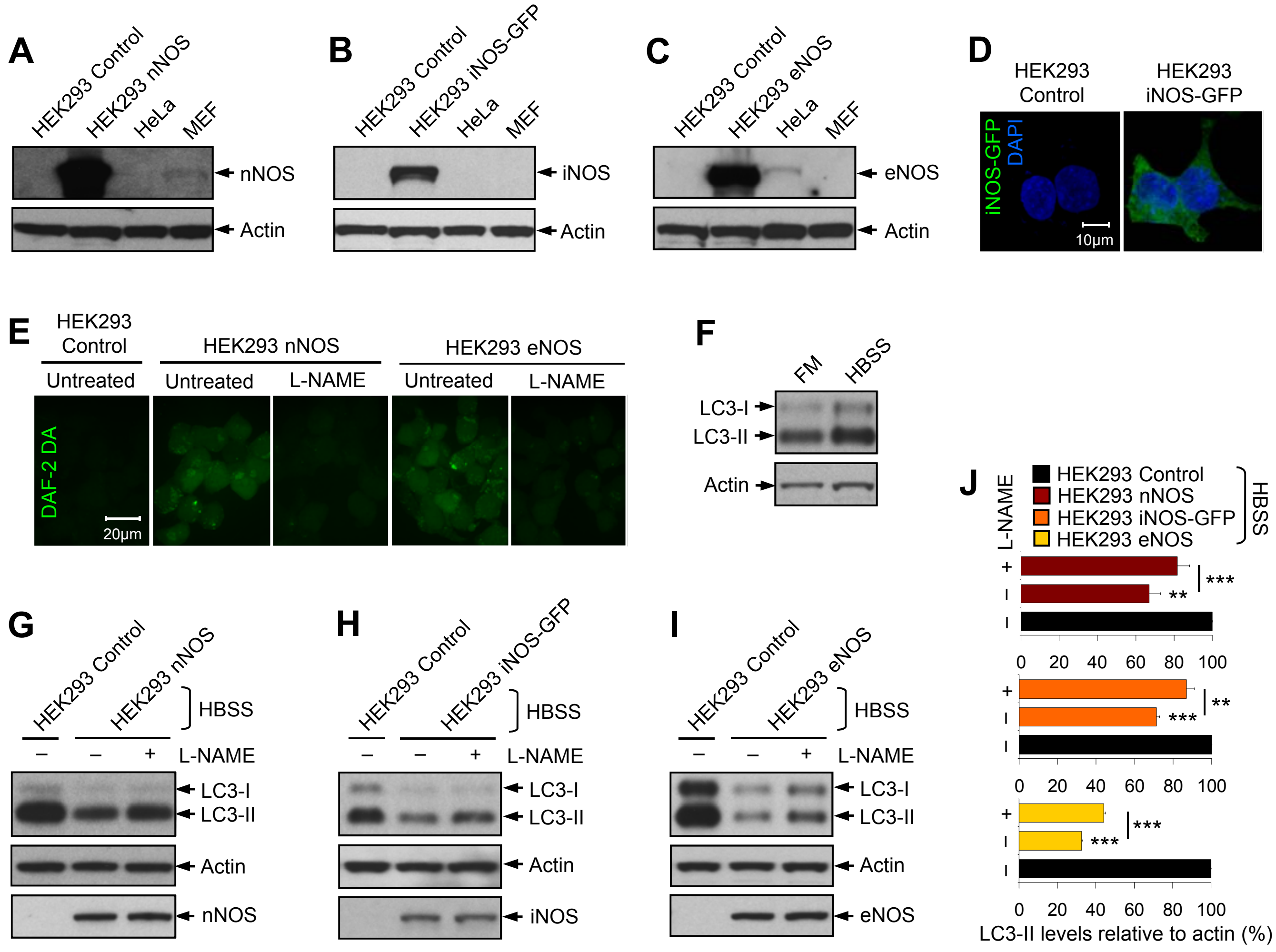


**Figure S5. Stable HEK293 Cell Lines Expressing nNOS, iNOS-GFP or eNOS Have Reduced Starvation-Induced Autophagy, which Is Partly Rescued by L-NAME**

**(A–C)** Immunoblot analyses with anti-nNOS (A), anti-iNOS (B) and anti-eNOS (C) antibodies confirm the expression of the respective NOS isoforms in stable HEK293 cell lines expressing nNOS (A), iNOS-GFP (B) or eNOS (C), respectively, and the absence of endogenous NOS isoforms in HEK293 control cells. Low levels of endogenous nNOS (A) and eNOS (C) were detected in MEFs (A) and HeLa cells (C), respectively.

**(D)** Confocal microscopy images show the expression of iNOS-GFP in stable HEK293 iNOS-GFP cell line. Scale bar, 10 μm.

**(E)** Confocal microscopy images show detection of nitric oxide (NO) production by DAF-2DA fluorescent dye in stable HEK293 cell lines expressing nNOS or eNOS (grown under $CaCl_2$ and L-arginine containing media) upon brief stimulation by ionomycin, whereas treatment of these cell lines with L-NAME prevented NO production as observed by reduced DAF-2DA fluorescence. No fluorescence was observed in HEK293 control cells due to the absence of endogenous NOS isoforms. Scale bar, 20 μm.

**(F)** Immunoblot analysis with anti-LC3 antibody show elevated LC3-II levels in HEK293 cells starved with Hank's Buffered Salt Solution (HBSS) compared to cells grown in full media (FM), confirming the induction of autophagy in these cells upon starvation.

**(G–J)** Immunoblot analyses with anti-LC3 antibody show that stable HEK293 cell lines expressing nNOS (G), iNOS-GFP (H) or eNOS (I) cultured under starvation condition with HBSS had significantly lower LC3-II levels compared to the HEK293 control cells ($p$=0.0051, nNOS; $p$<0.0001, iNOS-GFP; $p$<0.0001,eNOS) (J). Stable HEK293 cell lines treated with L-NAME had small but significant increase in LC3-II levels compared to the untreated nNOS ($p$=0.0002) (G and J), iNOS-GFP ($p$=0.0085) (H and J) or eNOS ($p$<0.0001) (I and J) expressing HEK293 cells. Note that immunoblotting analyses detecting endogenous LC3-II showed that L-NAME partly rescued the levels of autophagy in the stable HEK293 cell lines expressing NOS isoforms, whereas transient overexpression of cherry-LC3 under similar starvation condition showed a complete rescue by L-NAME as in Figures 5E and 5F. Graphical data are represented as mean +/– SEM. ***, $p$<0.001; **, $p$<0.01.

# FIGURE S6: Relates to Figure 6

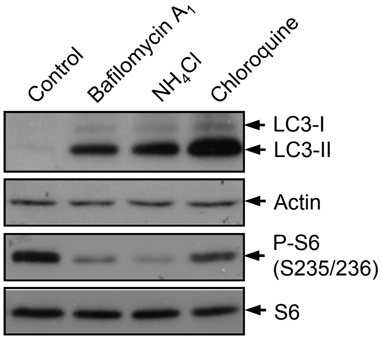


**Figure S6. Inhibition of Autophagosome-Lysosome Fusion Reduces mTOR Activity**
Immunoblot analyses with anti-LC3 and anti-phospho-S6 (Ser235/236) antibodies show that inhibition of autophagosome-lysosome fusion in HeLa cells, treated for 16 h with 200 nM bafilomycin $A_1$, 20 mM NH4Cl or 100 μM chloroquine (all autophagy blockers), increases LC3-II levels (by preventing LC3-II degradation) and reduces mTOR activity as assessed by phosphorylation of S6. Such an effect may explain the reduced mTOR activity (Figures 6A–6F) observed in HEK293 NOS stable cell lines that have impaired autophagy (Figures 5A–5H), which may result due to the cells being chronically starved with autophagy inhibition.

## FIGURE S7: Relates to Figure 7

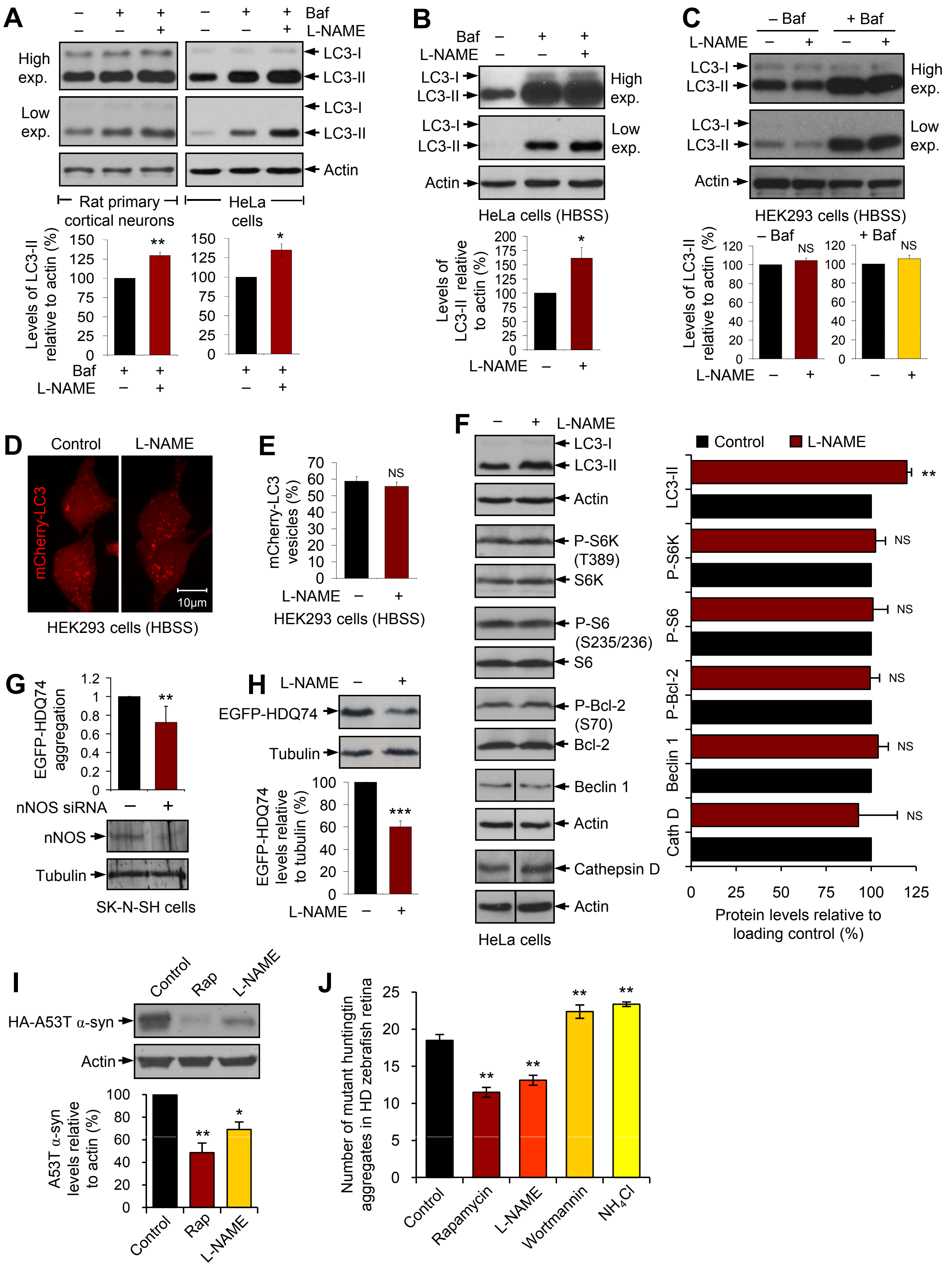

**Figure S7. L-NAME Induces Autophagy by Inhibiting Endogenous NOS, Independently of the Bcl-2 and mTOR Pathways, and Enhances the Clearance of Mutant Aggregate-prone Proteins**

**(A)** Immunoblot analysis with anti-LC3 antibody shows that L-NAME significantly increased autophagosome synthesis in bafilomycin $A_1$-treated rat primary cortical neurons ($p$=0.0027) and HeLa cells ($p$=0.0128), compared to bafilomycin $A_1$ treatment alone.

**(B)** Immunoblot analysis with anti-LC3 antibody shows that L-NAME-treated HeLa cells starved with Hank's Buffered Salt Solution (HBSS) significantly increased LC3-II levels in the presence of bafilomycin $A_1$, compared to bafilomycin $A_1$-treated cells alone ($p$=0.0119), suggesting that L-NAME stimulates autophagy under starvation condition. L-NAME stimulated autophagy under starvation condition also in HEK293 cell lines stably expressing nNOS, iNOS or eNOS (Figures 5E, 5F and S5G–S5J).

**(C)** Immunoblot analysis with anti-LC3 antibody shows that HEK293 cells (which do not have any endogenous NOS) treated with L-NAME had no significant effects on LC3-II levels and autophagosome synthesis in the absence ($p$=0.1638) or presence ($p$=0.1906) of bafilomycin $A_1$ compared to the untreated cells, suggesting that L-NAME induced autophagy by inhibiting endogenous NOS (as seen in L-NAME-treated NOS-overexpressing HEK293 stable cell lines in Figures 5A–5D).

**(D and E)** Confocal microscopy images (D) and assessment of mCherry-LC3 vesicles with a fluorescence microscope (E) show that *mCherry-LC3*–transfected HEK293 cells (which do not have any endogenous NOS) starved with HBSS and treated with L-NAME had no significant difference in mCherry-LC3 vesicles compared to untreated *mCherry-LC3* transfected cells ($p$=0.437), suggesting that the effect of L-NAME in stimulating autophagy under starvation condition is mediated by inhibiting endogenous NOS (as seen in NOS-overexpressing HEK293 cell lines in Figures 5E and 5F). Scale bar, 10 µm (D).

**(F)** Immunoblot analyses with anti-LC3, anti-phospho-S6K (Thr389), anti-phospho-S6 (Ser235/236), anti-phospho-Bcl-2 (Ser70), anti-beclin 1 and anti-cathepsin D antibodies show that HeLa cells treated with L-NAME had increased LC3-II levels ($p$=0.0015), but had no significant effects on S6K phosphorylation ($p$=0.6844), S6 phosphorylation ($p$=0.911), Bcl-2 phosphorylation ($p$=0.9189), beclin 1 levels ($p$=0.7713) and cathepsin D levels ($p$=0.7046) compared to the untreated cells, suggesting that induction of autophagy by inhibition of endogenous NOS is independent of the Bcl2 and mTORC1 pathways.

**(G)** Assessment of *EGFP-HDQ74*–transfected SK-N-SH cells with a fluorescence microscope shows that siRNA-mediated nNOS knockdown significantly reduced the proportion cells with EGFP-HDQ74 aggregates compared to the scrambled (control) siRNA-transfected cells ($p$<0.01). Immunoblot analysis with anti-nNOS antibody confirms a reduction in endogenous nNOS levels with nNOS siRNA.

**(H)** Immunoblot analysis with anti-EGFP antibody shows that stable inducible PC12 cell line expressing EGFP-HDQ74, induced with doxycycline and followed by treatment with L-NAME in the switch-off period, had significantly lower levels of soluble EGFP-HDQ74 compared to the untreated (control) cells ($p$<0.0001), suggesting that L-NAME enhanced the clearance of this autophagy substrate.

**(I)** Immunoblot analysis with anti-HA antibody shows that stable inducible PC12 cell line expressing HA-A53T α-synuclein, induced with doxycycline and followed by treatment with rapamycin ($p$=0.0011) or L-NAME ($p$=0.0131) in the switch-off period, had significantly lower levels of soluble HA-A53T α-synuclein compared to the untreated (control) cells, suggesting that both the autophagy inducers enhanced the clearance of this autophagy substrate.

**(J)** Transgenic Huntington's disease (HD) zebrafish expressing mutant huntingtin in the retina were treated with autophagy inducers like rapamycin and L-NAME, or with autophagy inhibitors like wortmanin (inhibitor of autophagosome formation) and $NH_4Cl$ (blocker of autophagosome-lysosome fusion). Treatment with rapamycin or L-NAME reduced the number of mutant huntingtin aggregates in the retina of transgenic HD zebrafish, whereas treatment with wortmannin or $NH_4Cl$ increased the number of aggregates ($p$<0.01 for all treatment conditions). Note that data in Figures 7H, 7I and S7J are from independent experiments.

Graphical data are represented as mean +/– SEM, except for Figure S7G, which is expressed as odds ratio with 95% confidence interval. ***, $p$<0.001; **, $p$<0.01; *, $p$<0.05; NS, Non-significant.

# SUPPLEMENTAL EXPERIMENTAL PROCEDURES

## Plasmids

*EGFP-HDQ74, mCherry-LC3 (mCherry-hLC3B), Flag-Beclin 1, hVps34, WT Bcl-2* and *empty Flag* constructs were described previously (Jahreiss et al., 2008; Luo and Rubinsztein, 2010; Narain et al., 1999; Sarkar et al., 2007b; Williams et al., 2008). Constructs received as kind gifts were *EGFP-LC3* and *mRFP-GFP-LC3* from T. Yoshimori (Kabeya et al., 2000; Kimura et al., 2007), *WT Myc-Bcl-2* and *AAA Myc-Bcl-2* from B. Levine (Wei et al., 2008), *Flag-JNK1* (*Flag-Jnk1a1*) and *Flag-CA JNK1* (*Flag-MKK7B2Jnk1a1*) from R.J. Davis (Addgene plasmids 13798 and 19726) (Derijard et al., 1994; Lei et al., 2002), *IKKβ-Flag* from W. Greene (Addgene plasmid 23298) (Geleziunas et al., 1998), *C229A Bcl-2* from Y. Rojanasakul (Azad et al., 2006) and *mCherry* from R. Tsien (Shaner et al., 2004).

## Chemical compounds

Compounds used in mammalian cell cultures were 200 nM rapamycin (Cat. No. R-5000, LC Laboratories), 400 nM bafilomycin $A_1$ (Cat. No. 19-148, Millipore), 500 μM DEA NONOate (Cat. No. D184), 500 μM DETA NONOate (Cat. No. A5581), 500 μM SIN-1 (Cat. No. M5793), 1 $\mu g.mL^{-1}$ doxycycline (Cat. No. D9891), 3 μM ionomycin (Cat. No. I0634), 100 μM chloroquine (Cat. No. C6628), 20 mM ammonium chloride ($NH_4Cl$) (Cat. No. A9434), 10 μM lactacystin (L6785) (all from Sigma-Aldrich), 100 μM L-NAME (Cat. No. 483125, Calbiochem), 1 mM 8-pCPT-cGMP (Cat. No. C009, Biolog) and 10 $ng.mL^{-1}$ TNFα (Cat. No. 315-01A, PeproTech). Cells were treated with NO donors (DEA NONOate, DETA NONOate, SIN-1), L-NAME, 8-pCPT-cGMP and rapamycin for 24 h, unless otherwise indicated. During the treatment with NO modulators, cells were replenished with fresh compounds thrice during the 24 h treatment period.

Compounds used for the treatment in Huntington's disease (HD) zebrafish model were 30 μM rapamycin (Cat. No. R-5000, LC Laboratories), 1 mM L-NAME (Cat. No. 483125, Calbiochem), 100 nM wortmannin (Cat. No. 681676, Calbiochem) or 10 mM ammonium chloride ($NH_4Cl$) (Cat. No. A9434, Sigma-Aldrich). Compound used for the treatment in HD *Drosophila* model was 1 mM L-NAME (Cat. No. N5751, Sigma-Aldrich).

## Mammalian cell culture and transfection

HeLa cells, HEK293 cells, SK-N-SH cells, $Atg5^{+/+}$ and $Atg5^{-/-}$ MEFs (kind gifts from N. Mizushima) (Kuma et al., 2004), *WT Bcl-2* and *AAA Bcl-2* MEFs (kind gifts from the laboratory of the late S.J. Korsmeyer) (Wei et al., 2008), *WT Jnk*, $Jnk1^{-/-}$ and $Jnk2^{-/-}$ MEFs (kind gifts from R.J. Davis) (Tournier et al., 2000), $Tsc2^{+/+}$ and $Tsc2^{-/-}$ MEFs (kind gifts from D.J. Kwiatkowski and A.R. Tee) (Zhang et al., 2003), $Ikk\beta^{+/+}$ and $Ikk\beta^{-/-}$ MEFs (kind gifts from M. Karin) (Hacker and Karin, 2006) were maintained in DMEM supplemented with 10% fetal bovine serum (FBS), 100 $U.mL^{-1}$ penicillin/streptomycin and 2 mM L-glutamine (all from Sigma-Aldrich) in 37°C, 5% $CO_2$ humidified incubator. HeLa cells stably expressing mRFP-GFP-LC3 reporter (HeLa mRFP-GFP-LC3 cells) (Sarkar et al., 2009a) were grown in the HeLa cell media supplemented with 600 $\mu g.mL^{-1}$ G418 (Invitrogen). HEK293 cells stably expressing nNOS (HEK293 nNOS; kind gift from S.H. Snyder) (Kornberg et al., 2010) were grown in the HEK293 cell media supplemented with 8 $\mu g.mL^{-1}$ tylosin (Sigma-Aldrich). HEK293 cells stably expressing iNOS-GFP (HEK293 iNOS-GFP; kind gift from N.T. Eissa) (Kolodziejska et al., 2005; Pandit et al., 2009) were grown in the HEK293 cell media supplemented with 600 $\mu g.mL^{-1}$ G418. HEK293 cells stably expressing eNOS (HEK293 eNOS; kind gift from W.C. Sessa) (Liu et al., 1996) were grown in the HEK293 cell media supplemented with 100 $\mu g.mL^{-1}$ G418. Stable inducible PC12 stable cell line expressing EGFP-HDQ74 (PC12 EGFP-HDQ74 cells) (Wyttenbach et al., 2001) or HA-A53T α-synuclein (PC12 HA-A53T α-synuclein cells) (Webb et al., 2003) were maintained at 75 $\mu g.mL^{-1}$ hygromycin B (Calbiochem) in DMEM with 10% horse serum, 5% FBS, 100 $U.mL^{-1}$ penicillin/streptomycin, 2 mM L-glutamine and 100 $\mu g.mL^{-1}$ G418 at 37°C, 10% $CO_2$.

Cells were transfected with DNA constructs for 4 h using Lipofectamine 2000 (Invitrogen) according to the manufacturer's protocol. The amount of DNA used during transfection in various experiments was as follows: 1.5 μg for *EGFP-HDQ74* and *Flag-CA JNK1*; 0.2 μg for *mCherry-LC3*; 0.5 μg for *EGFP-LC3*, except for Figure 4E where *pcDNA3.1* and *AAA Myc-Bcl-2* were used at 1.5 μg along with 0.5 μg of *EGFP-LC3* (3:1 ratio); 1 μg of each of the constructs in the immunoprecipitation experiments (1:1 ratio), except in Figure 2G where *pcDNA3.1* and *AAA Myc-Bcl-2* were used at 1.5 μg along with 0.75 μg of *hVps34*, *empty Flag* or *Flag-Beclin 1* (2:1:1 ratio) as indicated.

## Culture of rat, mouse and human cortical neurons

**Rat primary cortical neurons:** Primary cortical neurons were isolated from E16.5 Sprague Dawley rat pups (Charles River). Briefly, pup brains were harvested and placed in ice-cold DMEM where the meninges were removed and the cerebral cortices were dissected and incubated in DMEM. After mechanical dissociation using sterile micropipette tips, dissociated neurons were resuspended in DMEM and centrifuged. Cell count and viability assay was performed using the trypan blue exclusion test. Viable cells were seeded on poly-D-lysine and laminin-coated 6-multiwell plates (7.5 x $10^5$ cells per well). Cells were cultured in DMEM supplemented with 2 mM glutamine, 2% B27 supplement and 1% Penicillin-Streptomycin-Fungizone (PSF; Invitrogen) at 37°C in a humidified incubator with 5% $CO_2$ and 95% $O_2$. One-half of the plating medium was changed every third day until treatment. After 7 days of culturing *in vitro*, the differentiated cortical neurons were used for experiments.

**Mouse primary cortical neurons:** Mouse primary cortical neuronal cultures were prepared as described previously (Crossthwaite et al., 2002). Briefly, cortices were dissected from 15- to 16-day-old C57BL/6 mouse embryos and mechanically dissociated in PBS ($Ca^{2+}$- and $Mg^{2+}$-free) supplemented with glucose (33 mM). Neurons were plated into multi-well plates coated with 20 μg.mL$^{-1}$ poly-D-lysine and were maintained in DMEM, supplemented with B-27 (Gibco), 2 mM glutamine, 100 μg.mL$^{-1}$ streptomycin and 60 μg.mL$^{-1}$ penicillin, at 37°C and 5% $CO_2$. Cultures were used after 6–7 days *in vitro* when the majority of cells were neuronal and there were no detectable glial elements.
**Human cortical neurons:** ReNcell CX cells, an immortalized human neuronal progenitor cell line with the ability to readily differentiate into cortical neurons, were cultured according to the manufacturer's instructions (Chemicon, Millpore, MA, USA). Cells were grown in ReNcell NSC maintenance medium supplemented with 20 ng.mL$^{-1}$ bFGF and 20 ng.mL$^{-1}$ EGF. Cells were seeded on laminin coated plates and differentiation was initiated with media containing no growth factors for 1 week prior to starting the experiment.

## Immunoblot analysis

Cell pellets were lysed on ice in Laemmli buffer (62.5 mM Tris-HCl pH 6.8, 2% sodium dodecyl sulphate, 5% β-mercaptoethanol, 10% glycerol, and 0.01% bromophenol blue) for 30 min in presence of Complete protease inhibitor cocktail (Roche Diagnostics), boiled for 5-7 min at 100°C and subjected to western blot analysis, as previously described (Sarkar et al., 2009b). Primary antibodies include anti-LC3 (1:5,000 dilution; Cat. No. NB100-2220, Novus Biologicals), anti-phospho-Bcl-2 (Ser70) (Cat. No. 2827), anti-phospho-JNK (Thr183/Tyr185) (Cat. No. 9251), anti-p70S6 kinase (Cat. No. 9202), anti-phospho-p70S6 kinase (Thr389) (Cat. No. 9206), anti-S6 ribosomal protein (Cat. No. 2217), anti-phospho-S6 ribosomal protein (Ser235/236) (Cat. No. 2211), anti-TSC2 (Cat. No. 3990), anti-IKKα (Cat. No. 2682), anti-phospho-IKKα/β (Ser176/180) (Cat. No. 2687), anti-AMPKα (Cat. No. 2532), anti-phospho-AMPKα (Ser176/180) (Cat. No. 2531), anti-Beclin 1 (Cat. No. 3738), anti-Cathepsin D (Cat. No. 2284), anti-nNOS (Cat. No. 4231), anti-iNOS (Cat. No. 2977), anti-eNOS (Cat. No. 9586) (all 1:1,000 dilutions from Cell Signaling Technology), anti-Bcl-2 (1:2,000 dilution; Cat. No. sc-492), anti-JNK1 (1:250 dilution; Cat. No. sc-1648) (Santa Cruz Biotechnology), anti-Vps34 (1:500 dilution; Cat. No. 38-2100, Invitrogen), anti-EGFP (1:4,000 dilution; Cat. No. 632375, Clontech), anti-Myc (1:3,000 dilution; Cat. No. 11667203001, Roche Diagnostics), anti-Flag (1:2,000 dilution; Cat. No. F7425 or F3165), anti-HA (1:1,000 dilution; Cat. No. MMS-101R, Covance), rabbit anti-actin (1:4,000; Cat. No. A2066) and anti-tubulin (1:4,000 dilution; Cat. No. T6199) (Sigma-Aldrich). Blots were probed with anti-mouse or anti-rabbit IgG-HRP secondary antibody (1:4,000 dilutions) and visualised using ECL detection kit (GE Healthcare). TNFα and lactacystin treatment was done for 5 min for increasing the levels of detectable phospho-IKKα/β prior to harvesting the cells for immunoblot analysis.

## Analysis of autophagic flux in mRFP-GFP-LC3 HeLa cells with an automated Cellomics microscope

Assessment of autophagic flux involves the complete flow of the autophagosomes from their formation to fusion with the lysosomes. Counting of autolysosomes in stable HeLa cell line expressing mRFP-GFP-LC3 reporter was performed on a Thermo Scientific Cellomics ArrayScan HCS reader (20x objective) using the *Spot detector V3* Cellomics Bioapplication, as previously described (Sarkar et al., 2009a). In mRFP-GFP-LC3 HeLa cells, autophagosomes have both mRFP and GFP signals, whereas the autolysosomes emit only mRFP signal due to quenching of the GFP in the acidic lysosomal environment (Kimura et al., 2007). For identification of cells, nuclei were stained with DAPI and detected on the primary channel by a Hoechst-associated filter dependent upon size, shape, and intensity thresholding. GFP-LC3 and mRFP-LC3 vesicles were identified on separate secondary channels using FITC- and Texas Red-associated filters, respectively, dependent upon size, shape, and intensity thresholding. The mean number of vesicles per cell (object) was calculated by the ArrayScan software as the total number of vesicles per field divided by the total number of objects per field. The number of autolysosomes was analyzed by subtracting GFP dots (spot count per object by FITC-associated filter) from mRFP dots (spot count per object by Texas Red-associated filter). Cells were fixed with 4% formaldehyde and mounted on Citifluor containing DAPI prior to Cellomics analysis. 1,000 cells were counted per coverslip, and the analysis was done on triplicate samples at least twice.

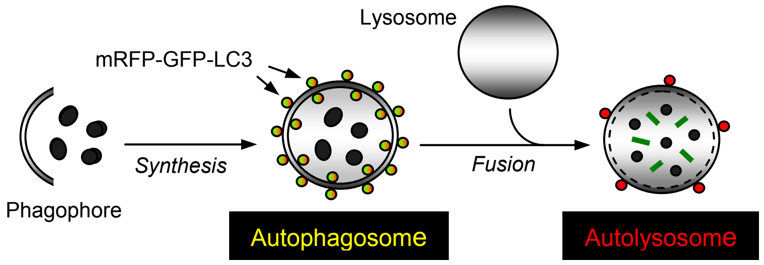


## Analysis of autophagosome synthesis by LC3-II levels in the presence of bafilomycin $A_1$

As the steady state levels of LC3-II are affected both by synthesis and degradation, effects on autophagy are best assessed by clamping LC3-II/autophagosome degradation with bafilomycin $A_1$. Bafilomycin $A_1$ inhibits the vacuolar $H^+$

ATPase (V-ATPase) and prevents acidification of autophagosomes and lysosomes, thereby inhibiting LC3-II degradation and resulting in increased LC3-II levels (Fass et al., 2006; Klionsky et al., 2008b; Yamamoto et al., 1998). This is an well established method for monitoring autophagosome synthesis (Klionsky et al., 2008a; Mizushima et al., 2010; Rubinsztein et al., 2009; Sarkar et al., 2007b; Sarkar et al., 2009b). An autophagy enhancer will further increase LC3-II levels in the presence of bafilomycin $A_1$, compared to bafilomycin $A_1$ alone, due to an increase in the synthesis of autophagosomes. In contrast, an autophagy inhibitor that prevents autophagosome formation will decrease LC3-II levels in the presence of bafilomycin $A_1$, compared to bafilomycin $A_1$ alone. Autophagosome synthesis was analysed by measuring the levels of LC3-II (immunoblotting with anti-LC3 antibody) in the presence of a saturating concentration (400 nM) of bafilomycin $A_1$, treated for the last 4 h prior to harvesting the cells for immunoblot analysis, as previously described (Rubinsztein et al., 2009; Sarkar et al., 2007a; Sarkar et al., 2007b; Sarkar et al., 2009b).

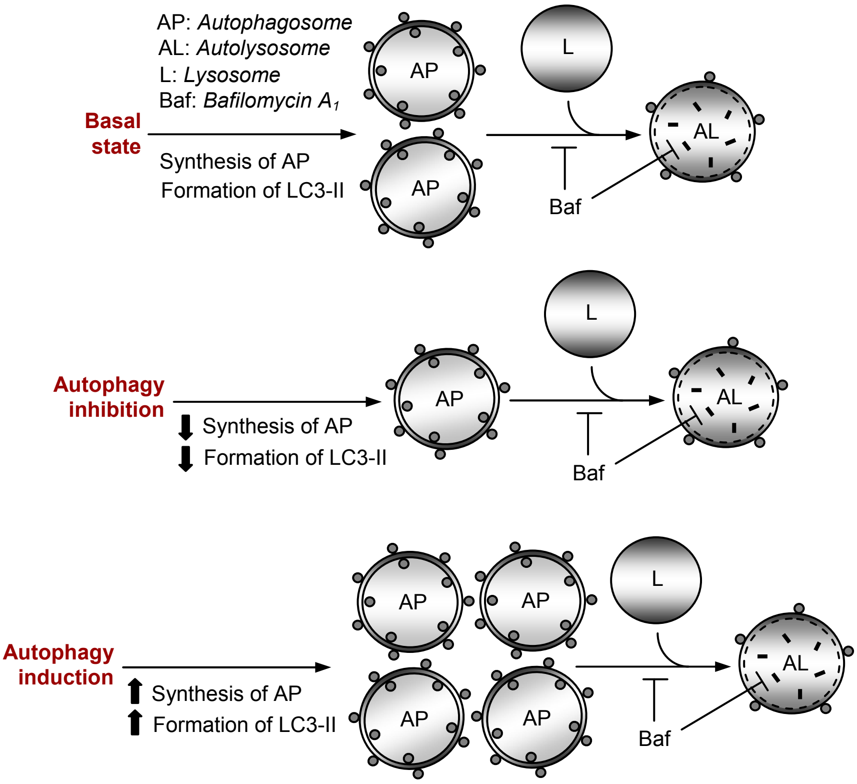


## Immunofluorescence analysis

Cells were washed with phosphate-buffered saline (PBS), fixed with 4% formaldehyde for 20 min at room temperature, then washed twice in PBS, followed by permeablization with 0.5% Triton in PBS for 10 min, and washed once more with PBS. Cells were then blocked in blocking buffer (5% FBS in PBS, or 2% BSA, 2% normal goat serum, 0.05% tween-20 in PBS) for 30 min at room temperature. Cells were incubated overnight at 4°C with primary antibodies, which include anti-Atg16 (1:100 dilution; Cat. No. PM040, MBL), anti-phospho-S6 ribosomal protein (Ser235/236) (1:200 dilution; Cat. No. 2211, Cell Signaling Technology), anti-LAMP1 (1:1500 dilution; Cat. No. H4A3, Developmental Studies Hybridoma Bank), anti-mTOR (1:100 dilution; Cat. No. 2972, Cell Signaling Technology), anti-phospho-mTOR (Ser2448) (1:100 dilution; Cat. No. 2971, Cell Signaling Technology). After washing three times in PBS, 10 min each, cells were incubated for 1 h at room temperature with secondary antibodies, which include Alexa Fluor 488 Green or Alexa Fluor 594 Red-conjugated antibodies (1:1000 dilutions; all raised in goat; Invitrogen). Cells were again washed three times in PBS, 10 min each. Cover slips were air-dried and then mounted on in Citifluor (Citifluor Ltd.) containing 3 $\mu g.mL^{-1}$ 4',6-diamidino-2-phenylindole (DAPI; Invitrogen). Images were acquired on a LSM510 META confocal microscope (Carl Zeiss Inc.), with 63x 1.4NA plan-apochromat oil immersion lens at room temperature, using Zeiss LSM510 v3.2 software. For detection of EGFP-LC3 vesicles or mRFP-GFP-LC3 vesicles, standard fluorescence method was used after fixing the cells with 4% formaldehyde and mounted on Citifluor containing DAPI, as described above.

## Immunoprecipitation

Cells were treated with NO donors in the last 24 h of the 48 h post-transfection period. Immunoprecipitation (IP) was performed using Buffer A (20 mM Tris-HCl pH 7.2, 2 mM $MgCl_2$, 150 mM NaCl, 0.5% NP-40 and Complete protease inhibitor cocktail). Cells were lysed in Buffer A for 30 min on ice, followed by centrifugation at 13,000 × *g* for 15 min. A small fraction of the cell lysates were aliquoted and stored (inputs), and the rest (500 µg to 1 mg protein) were incubated overnight at 4°C with anti-Flag M2 affinity agarose gel (Cat. No. A2220, Sigma) or anti-JNK1 agarose-conjugated beads (Cat. No. sc-1648-AC, Santa Cruz Biotechnology), added to a final concentration of 5 $\mu g.mL^{-1}$.

Following 3 washings in Buffer A, IP products were directly boiled in Laemmli buffer and subjected to immunoblot analysis, together with the inputs.

## Starvation of mammalian cells for autophagy induction

Cells were starved in Hanks' Balanced Salt Solution (HBSS) (Cat. No. H9394, Sigma) to induce autophagy. HeLa cells and HeLa mRFP-GFP-LC3 cells were cultured in HBSS for 6 h to stimulate starvation-induced autophagy. HEK293 cells, HEK293 nNOS cells, HEK293 iNOS-GFP cells and HEK293 eNOS cells were cultured in HBSS for 2 h for stimulating starvation-induced autophagy.

## Analysis of Atg16-positive structures

HeLa cells, HEK293 cells, HEK293 nNOS cells, HEK293 iNOS-GFP cells and HEK293 eNOS cells were starved in HBSS either for 6 h (HeLa cells) or 2 h (HEK293 cells and stable HEK293 NOS cells) prior to fixation with 4% formaldehyde and then subjected to immunofluorescence analysis with anti-Atg16 antibody, as described above. Cells were assessed with a fluorescence microscope for the percentage of cells containing Atg16-positive structures (phagophores). Images were acquired with a confocal microscope.

## Quantification of mutant huntingtin aggregates

The percentage of EGFP-positive cells with EGFP-HDQ74 aggregates was assessed with a fluorescence microscope, as previously described (Narain et al., 1999; Sarkar et al. 2005; Sarkar et al., 2007b; Sarkar et al., 2009b). *EGFP-HDQ74*-transfected cells were treated with compounds in the last 24 h of the 48 h post-transfection period.

## Analysis of clearance of soluble mutant huntingtin

Stable inducible PC12 cell line expressing EGFP-tagged mutant huntingtin exon 1 with 74 polyglutamine repeats (EGFP-HDQ74) (Wyttenbach et al., 2001) was induced with 1 μg.mL$^{-1}$ doxycycline (Sigma) for 8 h, followed by switching off the transgene expression by removing doxycycline from medium. We have previously shown that the clearance of EGFP-HDQ74 was accelerated upon autophagy induction, leading to lower levels of the transgene-encoded protein, compared to untreated cells after the switch-off period (Ravikumar et al., 2002; Sarkar et al. 2005; Sarkar et al., 2007a; Sarkar et al., 2009b; Williams et al., 2008). Cells were then treated with or without L-NAME for 120 h in the switch-off period, with the compound being replenished every 24 h. Clearance of soluble EGFP-HDQ74 was analysed by immunoblotting with anti-EGFP antibody.

## Analysis of A53T α-synuclein clearance

Stable inducible PC12 cell line expressing HA-tagged A53T mutant of α-synuclein (HA-A53T α-synuclein) (Webb et al., 2003) was induced with 1 μg.mL$^{-1}$ doxycycline (Sigma) for 48 h, followed by switching off the transgene expression by removing doxycycline from medium. We have previously shown that the clearance of HA-A53T α-synuclein was accelerated upon autophagy induction, leading to lower levels of the transgene-encoded protein, compared to untreated cells after the switch-off period (Sarkar et al. 2005; Sarkar et al., 2007a; Sarkar et al., 2009b; Webb et al., 2003; Williams et al., 2008). Cells were then treated with or without rapamycin or L-NAME for 24 h in the switch-off period. Clearance of HA-A53T α-synuclein was analysed by immunoblotting with anti-HA antibody.

## Biotin switch assay for *S*-nitrosylation

Detection of *S*-nitrosylated proteins was performed using a kit (Cat. No. 10006518, Cayman Chemical) that employs a modification of the previously described "biotin-switch" method (Jaffrey et al., 2001). Using this method, nitrosyl-groups are replaced with biotin residues. Biotinylated proteins were then precipitated with streptavidin-agarose beads (Cat. No. 20347, Thermo Scientific) and analysed by immunoblotting with anti-Flag antibody (1:2,000 dilution; Cat. No. F3165).

## Detection of nitric oxide production by DAF-2 DA fluorescence

DAF-2 DA (4,5-Diaminofluorescein diacetate; Cat. No. 85165, Cayman Chemical) is a sensitive fluorescent indicator for the detection and bioimaging of nitric oxide. It is the cell-permeable diacetate derivative of DAF-2 that is subsequently hydrolysed in cells by cytosolic esterases, releasing DAF-2, which is relatively non-fluorescent at physiological pH. Generation of nitric oxide in cells converts DAF-2 to the fluorescent triazole derivative, DAF-2T, which can be detected with excitation/emission maxima of 485/515 nm, respectively (Kojima et al., 1998; Kojima et al., 1999). HEK293 cells stably expressing nNOS or eNOS were cultured in HBSS for 1 h followed by addition of 100 μM L-Arginine and 1.3 mM $CaCl_2$ for an additional 30 min. Cells were then loaded with 10 μM DAF-2 DA for 15 min, followed by stimulation of NOS activity with 3 μM Ionomycin for another 15 min. Cells were fixed with 4% paraformaldehyde and images were acquired by a confocal microscope.

## RNA Interference

*SMART*pool siRNA (pool of four different siRNA duplexes) against human nNOS (Dharmacon) were used for knockdown of nNOS. *SMART*pool control siRNA (Dharmacon), which does not target any human or mouse genes,

was used as a control. SK-N-SH cells were transfected with 100 nM *SMART*pool siRNAs for 72 h using Oligofectamine (Invitrogen) according to manufacturer's protocol. Knockdown of nNOS was confirmed by immunoblotting with anti-nNOS antibody.

## Analysis of neurodegeneration in a *Drosophila* model of Huntington's disease

Pseudopupil analysis was performed, as previously described (Franceschini, 1972), by crossing virgin flies of the genotype *y w; P{GMR-HD.Q120}2.4* (Q120) (Jackson et al., 1998) with $w^{1118}$ isogenic males (Ryder et al., 2004). The progeny of the above cross was reared on instant fly food (Philip Harris Ltd.) containing 1 mM L-NAME. Control flies grown in absence of the drug were fed with instant fly food with 0.02% DMSO (Sigma). Flies were treated with drugs both during larval and adult stage. To assess the effect of L-NAME on Q120 flies, *t*-test analysis was performed on approximately 30 flies, 3 days post-eclosion. Each experiment was based on 4 independent sessions, where 15 ommatidia were scored from approximately 10 individuals of the same genotype. Genetic inhibition of NOS was done by crossing virgin flies of the above genotype to males *w; P{GMR-Nos.4.FLAG}* (NOS4) (Stasiv et al., 2004). The progeny of Q120 and NOS4 virgins crossed with $w^{1118}$ isogenic males (Ryder et al., 2004) was used as a control to assess level of neurodegeneration due to Q120 and NOS4 expression. Each experiment was based on 4 independent sessions, where 15 ommatidia were scored from approximately 15 individuals of the same genotype. Fly crosses and experiments were performed at 25°C. All crosses for individual experiments were performed at the same time and under the same conditions.

## Maintenance of zebrafish stocks and collection of embryos

Zebrafish were reared under standard conditions on a 14 h light:10 h dark cycle (Westerfield, 1995). Embryos were collected from natural spawnings, staged according to established criteria (Kimmel et al., 1995) and reared in embryo medium (5 mM NaCl, 0.17 mM KCl, 0.33 mM $CaCl_2$, 0.33 mM $Mg_2SO_4$, 5 mM HEPES). Aggregate counting was performed using the heterozygous larvae from the Tg(rho:EGFP-HTT71Q)$^{cu5}$ zebrafish line (Williams et al., 2008) (hereafter referred to as transgenic HD zebrafish). Compound exposure experiments were performed in the dark at 28.5 °C.

## Determination of the maximum tolerated concentration of compounds in larval zebrafish

Compound exposure experiments were performed on wild-type larvae (TL strain) from 3 days post-fertilization (d.p.f.) to 7 d.p.f. Concentration response assays were performed over half-log intervals, from 1 µM – 100 µM for rapamycin, 1 µM – 1 mM for L-NAME, 1 mM – 300 mM for ammonium chloride ($NH_4Cl$) and 10 nM – 3 µM for wortmannin, to determine the maximum non-toxic concentration for subsequent aggregate count assays (n = 10 larvae per concentration). The media and compounds were replenished every 24 h.

## Mutant huntingtin aggregate analysis in a transgenic Huntington's disease zebrafish model

All zebrafish experiments have been approved by local ethics committee and performed under the appropriate Home Office Licences. Embryos from out-crossed transgenic HD zebrafish were raised in 0.2 mM 1-phenyl-2-thiourea (PTU) from 1 to 3 days post-fertilization (d.p.f.) to inhibit pigment formation, screened for transgene expression using EGFP fluorescence then washed twice in embryo medium to remove PTU. From 3 d.p.f. to 8 d.p.f., transgenic HD zebrafish larvae were dark-reared in embryo medium alone or embryo medium containing either 30 µM rapamycin, 1 mM L-NAME, 100 nM wortmannin or 10 mM ammonium chloride ($NH_4Cl$); 1 mM L-NAME and 10 mM $NH_4Cl$ for combination treatment. Embryo medium and compounds were replenished daily. At 8 d.p.f., larvae were anesthetized by immersion in 0.2 $mg.mL^{-1}$ 3-amino benzoic acid ethylester (MS222) then fixed using 4% paraformaldehyde in PBS at 4 °C. Larvae were washed briefly in PBS, allowed to equilibrate in 30% sucrose in PBS then embedded in OCT medium (Tissue-Tek) and frozen on dry ice for subsequent cryosectioning. Sections were cut at 10 µm thickness using a Leica CM3050 cryostat and mounted in 80% glycerol in PBS. The total number of GFP-positive aggregates were counted over 100 µm of the central retina, either side of the optic nerve using a Zeiss Axiophot2 microscope and mean values were calculated using 6 fish (12 eyes) for each treatment group. Data were compared using ANOVA and Dunnetts post-hoc T-test. Representative images were taken using a Sony CCD digital camera.

## Statistical analyses

Densitometric analysis on the immunoblots was done by Image J software. The *p* values for densitometric analysis and Cellomics data were determined by factorial ANOVA test using STATVIEW v4.53 (Abacus Concepts), where the control condition was set to 100% and the data represented as mean ± standard error of mean (SEM). The y-axis values are shown in percentage (%). Experiments were performed in triplicates at least twice. The *p* values for assessing EGFP-HDQ74 aggregation, EGFP-LC3 or mCherry-LC3 vesicles were determined by unconditional logistical regression analysis, using the general log-linear analysis option of SPSS 9 software (SPSS, Chicago), as previously described (Ravikumar et al., 2008; Sarkar et al., 2007a; Sarkar et al., 2007b; Sarkar et al., 2009b; Williams et al., 2008). ***, $p < 0.001$; **, $p < 0.01$; *, $p < 0.05$; NS, Non-significant.

## SUPPLEMENTAL REFERENCES

Azad, N., Vallyathan, V., Wang, L., Tantishaiyakul, V., Stehlik, C., Leonard, S.S., and Rojanasakul, Y. (2006). S-nitrosylation of Bcl-2 inhibits its ubiquitin-proteasomal degradation. A novel antiapoptotic mechanism that suppresses apoptosis. J. Biol. Chem. *281*, 34124-34134.

Crossthwaite, A.J., Hasan, S., and Williams, R.J. (2002). Hydrogen peroxide-mediated phosphorylation of ERK1/2, Akt/PKB and JNK in cortical neurones: dependence on Ca(2+) and PI3-kinase. J. Neurochem. *80*, 24-35.

Derijard, B., Hibi, M., Wu, I.H., Barrett, T., Su, B., Deng, T., Karin, M., and Davis, R.J. (1994). JNK1: a protein kinase stimulated by UV light and Ha-Ras that binds and phosphorylates the c-Jun activation domain. Cell *76*, 1025-1037.

Fass, E., Shvets, E., Degani, I., Hirschberg, K., and Elazar, Z. (2006). Microtubules support production of starvation-induced autophagosomes but not their targeting and fusion with lysosomes. J. Biol. Chem. *281*, 36303-36316.

Franceschini, N. (1972). Pupil and pseudopupil in the compound eye of *Drosophila*. In Information processing in the visual system of *Drosophila*, R. Wehner, ed. (Berlin: Springer), pp. 75-82.

Geleziunas, R., Ferrell, S., Lin, X., Mu, Y., Cunningham, E.T., Jr., Grant, M., Connelly, M.A., Hambor, J.E., Marcu, K.B., and Greene, W.C. (1998). Human T-cell leukemia virus type 1 Tax induction of NF-kappaB involves activation of the IkappaB kinase alpha (IKKalpha) and IKKbeta cellular kinases. Mol. Cell Biol. *18*, 5157-5165.

Hacker, H., and Karin, M. (2006). Regulation and function of IKK and IKK-related kinases. Sci. STKE *2006*, re13.

Jackson, G.R., Salecker, I., Dong, X., Yao, X., Arnheim, N., Faber, P.W., MacDonald, M.E., and Zipursky, S.L. (1998). Polyglutamine-expanded human huntingtin transgenes induce degeneration of Drosophila photoreceptor neurons. Neuron *21*, 633-642.

Jaffrey, S.R., Erdjument-Bromage, H., Ferris, C.D., Tempst, P., and Snyder, S.H. (2001). Protein S-nitrosylation: a physiological signal for neuronal nitric oxide. Nat. Cell Biol. *3*, 193-197.

Jahreiss, L., Menzies, F.M., and Rubinsztein, D.C. (2008). The itinerary of autophagosomes: from peripheral formation to kiss-and-run fusion with lysosomes. Traffic *9*, 574-587.

Kabeya, Y., Mizushima, N., Ueno, T., Yamamoto, A., Kirisako, T., Noda, T., Kominami, E., Ohsumi, Y., and Yoshimori, T. (2000). LC3, a mammalian homologue of yeast Apg8p, is localized in autophagosome membranes after processing. EMBO J. *19*, 5720-5728.

Kimmel, C.B., Ballard, W.W., Kimmel, S.R., Ullmann, B., and Schilling, T.F. (1995). Stages of embryonic development of the zebrafish. Dev. Dyn. *203*, 253-310.

Kimura, S., Noda, T., and Yoshimori, T. (2007). Dissection of the autophagosome maturation process by a novel reporter protein, tandem fluorescent-tagged LC3. Autophagy *3*, 452-460.

Klionsky, D.J., Abeliovich, H., Agostinis, P., Agrawal, D.K., Aliev, G., Askew, D.S., Baba, M., Baehrecke, E.H., Bahr, B.A., Ballabio, A.*, et al.* (2008a). Guidelines for the use and interpretation of assays for monitoring autophagy in higher eukaryotes. Autophagy *4*, 151-175.

Klionsky, D.J., Elazar, Z., Seglen, P.O., and Rubinsztein, D.C. (2008b). Does bafilomycin A1 block the fusion of autophagosomes with lysosomes? Autophagy *4*, 849-950.

Kojima, H., Nakatsubo, N., Kikuchi, K., Kawahara, S., Kirino, Y., Nagoshi, H., Hirata, Y., and Nagano, T. (1998). Detection and imaging of nitric oxide with novel fluorescent indicators: diaminofluoresceins. Anal. Chem. *70*, 2446-2453.

Kojima, H., Urano, Y., Kikuchi, K., Higuchi, T., Hirata, Y., and Nagano, T. (1999). Fluorescent Indicators for Imaging Nitric Oxide Production. Angew. Chem. Int. Ed. Engl. *38*, 3209-3212.

Kolodziejska, K.E., Burns, A.R., Moore, R.H., Stenoien, D.L., and Eissa, N.T. (2005). Regulation of inducible nitric oxide synthase by aggresome formation. Proc. Natl. Acad. Sci. USA *102*, 4854-4859.

Kornberg, M.D., Sen, N., Hara, M.R., Juluri, K.R., Nguyen, J.V., Snowman, A.M., Law, L., Hester, L.D., and Snyder, S.H. (2010). GAPDH mediates nitrosylation of nuclear proteins. Nat. Cell Biol. *12*, 1094-1100.

Kuma, A., Hatano, M., Matsui, M., Yamamoto, A., Nakaya, H., Yoshimori, T., Ohsumi, Y., Tokuhisa, T., and Mizushima, N. (2004). The role of autophagy during the early neonatal starvation period. Nature *432*, 1032-1036.

Lei, K., Nimnual, A., Zong, W.X., Kennedy, N.J., Flavell, R.A., Thompson, C.B., Bar-Sagi, D., and Davis, R.J. (2002). The Bax subfamily of Bcl2-related proteins is essential for apoptotic signal transduction by c-Jun NH(2)-terminal kinase. Mol. Cell Biol. *22*, 4929-4942.

Liu, J., Garcia-Cardena, G., and Sessa, W.C. (1996). Palmitoylation of endothelial nitric oxide synthase is necessary for optimal stimulated release of nitric oxide: implications for caveolae localization. Biochemistry *35*, 13277-13281.

Luo, S., and Rubinsztein, D.C. (2010). Apoptosis blocks Beclin 1-dependent autophagosome synthesis: an effect rescued by Bcl-xL. Cell Death Differ. *17*, 268-277.

Mizushima, N., Yoshimori, T., and Levine, B. (2010). Methods in mammalian autophagy research. Cell *140*, 313-326.

Narain, Y., Wyttenbach, A., Rankin, J., Furlong, R.A., and Rubinsztein, D.C. (1999). A molecular investigation of true dominance in Huntington's disease. J. Med. Genet. *36*, 739-746.

Pandit, L., Kolodziejska, K.E., Zeng, S., and Eissa, N.T. (2009). The physiologic aggresome mediates cellular inactivation of iNOS. Proc. Natl. Acad. Sci. USA *106*, 1211-1215.

Ravikumar, B., Duden, R., and Rubinsztein, D.C. (2002). Aggregate-prone proteins with polyglutamine and polyalanine expansions are degraded by autophagy. Hum. Mol. Genet. *11*, 1107-1117.

Ravikumar, B., Sarkar, S., and Rubinsztein, D.C. (2008). Clearance of mutant aggregate-prone proteins by autophagy. Methods Mol. Biol. *445*, 195-211.

Rubinsztein, D.C., Cuervo, A.M., Ravikumar, B., Sarkar, S., Korolchuk, V., Kaushik, S., and Klionsky, D.J. (2009). In search of an "autophagomometer". Autophagy *5*, 585-589.

Ryder, E., Blows, F., Ashburner, M., Bautista-Llacer, R., Coulson, D., Drummond, J., Webster, J., Gubb, D., Gunton, N., Johnson, G.*, et al.* (2004). The DrosDel collection: a set of P-element insertions for generating custom chromosomal aberrations in Drosophila melanogaster. Genetics *167*, 797-813.

Sarkar, S., Davies, J.E., Huang, Z., Tunnacliffe, A., and Rubinsztein, D.C. (2007a). Trehalose, a novel mTOR-independent autophagy enhancer, accelerates the clearance of mutant huntingtin and alpha-synuclein. J. Biol. Chem. *282*, 5641-5652.

Sarkar, S., Floto, R.A., Berger, Z., Imarisio, S., Cordenier, A., Pasco, M., Cook, L.J., and Rubinsztein, D.C. (2005). Lithium induces autophagy by inhibiting inositol monophosphatase. J. Cell Biol. *170*, 1101-1111.

Sarkar, S., Korolchuk, V., Renna, M., Winslow, A., and Rubinsztein, D.C. (2009a). Methodological considerations for assessing autophagy modulators: a study with calcium phosphate precipitates. Autophagy *5*, 307-313.

Sarkar, S., Perlstein, E.O., Imarisio, S., Pineau, S., Cordenier, A., Maglathlin, R.L., Webster, J.A., Lewis, T.A., O'Kane, C.J., Schreiber, S.L., and Rubinsztein, D.C. (2007b). Small molecules enhance autophagy and reduce toxicity in Huntington's disease models. Nat. Chem. Biol. *3*, 331-338.

Sarkar, S., Ravikumar, B., and Rubinsztein, D.C. (2009b). Autophagic clearance of aggregate-prone proteins associated with neurodegeneration. Methods Enzymol. *453*, 83-110.

Shaner, N.C., Campbell, R.E., Steinbach, P.A., Giepmans, B.N., Palmer, A.E., and Tsien, R.Y. (2004). Improved monomeric red, orange and yellow fluorescent proteins derived from Discosoma sp. red fluorescent protein. Nat. Biotechnol. *22*, 1567-1572.

Stasiv, Y., Kuzin, B., Regulski, M., Tully, T., and Enikolopov, G. (2004). Regulation of multimers via truncated isoforms: a novel mechanism to control nitric-oxide signaling. Genes Dev. *18*, 1812-1823.

Tournier, C., Hess, P., Yang, D.D., Xu, J., Turner, T.K., Nimnual, A., Bar-Sagi, D., Jones, S.N., Flavell, R.A., and Davis, R.J. (2000). Requirement of JNK for stress-induced activation of the cytochrome c-mediated death pathway. Science *288*, 870-874.

Webb, J.L., Ravikumar, B., Atkins, J., Skepper, J.N., and Rubinsztein, D.C. (2003). Alpha-synuclein is degraded by both autophagy and the proteasome. J. Biol. Chem. *278*, 25009-25013.

Wei, Y., Pattingre, S., Sinha, S., Bassik, M., and Levine, B. (2008). JNK1-mediated phosphorylation of Bcl-2 regulates starvation-induced autophagy. Mol. Cell *30*, 678-688.

Westerfield, M. (1995). The Zebrafish Book: A Guide for the Laboratory Use of Zebrafish (Danio Rerio) (Eugene, Oregon: University of Oregon Press).

Williams, A., Sarkar, S., Cuddon, P., Ttofi, E.K., Saiki, S., Siddiqi, F.H., Jahreiss, L., Fleming, A., Pask, D., Goldsmith, P.*, et al.* (2008). Novel targets for Huntington's disease in an mTOR-independent autophagy pathway. Nat. Chem. Biol. *4*, 295-305.

Wyttenbach, A., Swartz, J., Kita, H., Thykjaer, T., Carmichael, J., Bradley, J., Brown, R., Maxwell, M., Schapira, A., Orntoft, T.F.*, et al.* (2001). Polyglutamine expansions cause decreased CRE-mediated transcription and early gene expression changes prior to cell death in an inducible cell model of Huntington's disease. Hum. Mol. Genet. *10*, 1829-1845.

Yamamoto, A., Tagawa, Y., Yoshimori, T., Moriyama, Y., Masaki, R., and Tashiro, Y. (1998). Bafilomycin A1 prevents maturation of autophagic vacuoles by inhibiting fusion between autophagosomes and lysosomes in rat hepatoma cell line, H-4-II-E cells. Cell Struct. Funct. *23*, 33-42.

Zhang, H., Cicchetti, G., Onda, H., Koon, H.B., Asrican, K., Bajraszewski, N., Vazquez, F., Carpenter, C.L., and Kwiatkowski, D.J. (2003). Loss of Tsc1/Tsc2 activates mTOR and disrupts PI3K-Akt signaling through downregulation of PDGFR. J. Clin. Invest. *112*, 1223-1233.

# SUPPLEMENTAL NOTES
## STATISTICAL ANALYSES DATA (*P* VALUES) OF FIGURES 1–7

## FIGURE 1
**(A)** Rat primary cortical neurons – DEA NONOate: $p$=0.0224; DETA NONOate: $p$=0.042; SIN-1: $p$=0.0002.
Bafilomycin $A_1$-treated rat primary cortical neurons – DEA NONOate: $p$=0.0009; DETA NONOate: $p$=0.0009; SIN-1: $p$=0.0004.
Bafilomycin $A_1$-treated HeLa cells – DEA NONOate: $p$=0.0224; DETA NONOate: $p$=0.042; SIN-1: $p$=0.0002.
**(B)** mRFP-GFP LC3 HeLa cells – DEA NONOate: $p$=0.0011; DETA NONOate: $p$=0.0007.
**(C)** mRFP-GFP LC3 HeLa cells – DEA NONOate: $p$=0.0206; DETA NONOate: $p$=0.0001; Untreated FM vs Untreated HBSS: $p$=0.0013.
**(F)** $Atg5^{+/+}$ MEFs – DEA NONOate: $p$=0.979 (100 μM), $p$=0.002 (500 μM); DETA NONOate: $p$=0.012 (100 μM), $p$=0.004 (500 μM); SIN-1: $p$=0.979 (100 μM), $p$=0.001 (500 μM).
$Atg5^{-/-}$ MEFs – DEA NONOate: $p$=0.802 (100 μM), $p$=0.8 (500 μM); DETA NONOate: $p$=0.802 (100 μM), $p$=0.886 (500 μM); SIN-1: $p$=0.942 (100 μM), $p$=0.516 (500 μM); Control $Atg5^{+/+}$ MEFs vs Control $Atg5^{-/-}$ MEFs: $p$<0.0001.
**(G)** HeLa cells – DEA NONOate: $p$<0.0001; DETA NONOate: $p$<0.0001.

## FIGURE 2
**(A)** HeLa cells – DEA NONOate: $p$=0.0018; DETA NONOate: $p$=0.0019.
**(B)** HeLa cells – DEA NONOate: $p$=0.0305; DETA NONOate: $p$=0.0082.
**(C)** HeLa cells – pcDNA3.1-transfected cells vs *Flag-CA JNK1*-transfected cells: $p$=0.0039; *Flag-CA JNK1*-transfected cells vs DETA NONOate-treated *Flag-CA JNK1*-transfected cells: $p$=0.1951.
**(D)** HeLa cells – DEA NONOate: $p$=0.0043, DETA NONOate: $p$=0.0132.
**(E)** HeLa cells – DEA NONOate: $p$=0.9079, DETA NONOate: $p$=0.8109.
**(F)** HeLa cells – DEA NONOate: $p$=0.0304, DETA NONOate: $p$=0.0311.
**(G)** HeLa cells – AAA Myc-Bcl-2: $p$=0.0366 (left panel); DETA NONOate + pcDNA3.1: $p$=0.0006 (right panel); AAA Myc-Bcl-2: $p$=0.0003 (right panel); DETA NONOate + AAA Myc-Bcl-2: $p$=0.0002 (right panel); AAA Myc-Bcl-2 vs DETA NONOate + AAA Myc-Bcl-2: $p$=0.5452 (right panel).

## FIGURE 3
**(A)** *WT Bcl-2* MEFs – DETA NONOate: $p$=0.0077.
*AAA Bcl-2* MEFs – DETA NONOate: $p$=0.024.
**(C)** *WT Bcl-2* MEFs – DEA NONOate: $p$<0.0001; DETA NONOate: $p$<0.0001.
*AAA Bcl-2* MEFs – DEA NONOate: $p$<0.0001; DETA NONOate: $p$<0.0001.
**(D)** *WT Bcl-2* MEFs – DEA NONOate: $p$<0.0001; DETA NONOate: $p$<0.0001.
*AAA Bcl-2* MEFs – DEA NONOate: $p$=0.006; DETA NONOate: $p$=0.027.
**(E)** *WT Jnk* MEFs – DETA NONOate: $p$=0.0048.
$Jnk1^{-/-}$ MEFs – DETA NONOate: $p$=0.0018.
$Jnk2^{-/-}$ MEFs – DETA NONOate: $p$=0.0005.
**(F)** HeLa cells – DEA NONOate: $p$<0.0001 (P-S6K), $p$=0.0014 (P-S6); DETA NONOate: $p$<0.0001 (P-S6K), $p$=0.0008 (P-S6).
**(H)** *WT Bcl-2* MEFs – DEA NONOate $p$=0.0105 (P-S6K), $p$=0.0001 (P-S6); DETA NONOate: $p$=0.0058 (S6K), $p$<0.0001 (P-S6).
*AAA Bcl-2* MEFs – DEA NONOate: $p$=0.0122 (P-S6K), $p$=0.001 (P-S6); DETA NONOate: $p$=0.0023 (P-S6K), $p$=0.0001 (P-S6).

## FIGURE 4
**(A)** $Tsc2^{+/+}$ MEFs – DEA NONOate: $p$=0.0094 (P-S6K), $p$=0.0243 (P-S6); DETA NONOate $p$=0.0019 (P-S6K), $p$=0.0256 (P-S6).
$Tsc2^{-/-}$ MEFs – DEA NONOate: $p$=0.6355 (P-S6K), $p$=0.7035 (P-S6); DETA NONOate $p$=0.8799 (P-S6K), $p$=0.2561 (P-S6).
**(B)** MEFs – DEA NONOate: $p$<0.0001 (P-IKKα/β), $p$<0.0001 (P-AMPKα); DETA NONOate: $p$<0.0001 (P-IKKα/β), $p$<0.0001 (P-AMPKα).
**(C)** $Ikk\beta^{+/+}$ MEFs – DEA NONOate: $p$<0.0001 (P-S6K), $p$=0.0053 (P-S6); DETA NONOate $p$<0.0001 (P-S6K), $p$=0.0009 (P-S6).
$Ikk\beta^{-/-}$ MEFs – DEA NONOate: $p$=0.22 (P-S6K), $p$=0.527 (P-S6); DETA NONOate $p$=0.539 (P-S6K), $p$=0.1905 (P-S6).
**(D)** $Tsc2^{+/+}$ MEFs – DETA NONOate: $p$=0.0009.
$Tsc2^{-/-}$ MEFs – DETA NONOate: $p$=0.007.
**(E)** $Tsc2^{+/+}$ MEFs – pcDNA3.1 + DETA NONOate: $p$<0.0001; AAA Myc-Bcl-2: $p$<0.0001; AAA Myc-Bcl-2 + DETA NONOate: $p$<0.0001; pcDNA3.1 + DETA NONOate vs AAA Myc-Bcl-2 + DETA NONOate: $p$=0.023.

*Tsc2*$^{-/-}$ MEFs – pcDNA3.1 + DETA NONOate: $p$=0.031; AAA Myc-Bcl-2: $p$=0.002; AAA Myc-Bcl-2 + DETA NONOate: $p$=0.011; pcDNA3.1 + DETA NONOate *Tsc2*$^{-/-}$ MEFs vs AAA Myc-Bcl-2 + DETA NONOate *Tsc2*$^{-/-}$ MEFs: $p$=0.862.

## FIGURE 5

**(D)** LC3 levels in the absence of bafilomycin $A_1$ – HEK293 nNOS cells: $p$=0.0001; HEK293 iNOS-GFP cells: $p$<0.0001; HEK293 eNOS cells: $p$<0.0001.
LC3 levels in the presence of bafilomycin $A_1$ – HEK293 nNOS cells: $p$<0.0001; HEK293 iNOS-GFP cells: $p$=0.0001; HEK293 eNOS cells: $p$<0.0001.
**(F)** mCherry-LC3 vesicles – HEK293 nNOS cells: $p$<0.0001; HEK293 iNOS-GFP cells: $p$<0.0001; HEK293 eNOS cells: $p$<0.0001;
mCherry-LC3 vesicles in the presence of L-NAME – HEK293 nNOS cells: $p$=0.002; HEK293 iNOS-GFP cells: $p$<0.0001; HEK293 eNOS cells: $p$<0.0001.
**(H)** Atg16-positive structures – HEK293 nNOS cells: $p$<0.0001; HEK293 iNOS-GFP cells: $p$<0.0001; HEK293 eNOS cells: $p$<0.0001.

## FIGURE 6

**(A)** HEK293 nNOS cells (FM) – P-JNK1: $p$=0.0002; P-Bcl-2: $p$=0.0008; P-S6K: $p$<0.0001.
**(B)** HEK293 iNOS-GFP cells (FM) – P-JNK1: $p$=0.0002; P-Bcl-2: $p$=0.0033; P-S6K: $p$<0.0001.
**(C)** HEK293 eNOS cells (FM) – P-JNK1: $p$=0.0003; P-Bcl-2: $p$<0.0001; P-S6K: $p$<0.0001.
**(D)** HEK293 nNOS cells (HBSS) – P-JNK1: $p$=0.0017; P-Bcl-2: $p$=0.0002; P-S6: $p$<0.0001.
**(E)** HEK293 iNOS-GFP cells (HBSS) – P-JNK1: $p$=0.0004; P-Bcl-2: $p$=0.0011; P-S6: $p$<0.0001.
**(F)** HEK293 eNOS cells (HBSS) – P-JNK1: $p$=0.0009; P-Bcl-2: $p$<0.0001; P-S6: $p$<0.0001.

## FIGURE 7

**(A)** Rat primary cortical neurons – Rapamycin: $p$=0.0011; L-NAME: $p$=0.0159.
HeLa cells – Rapamycin: $p$=0.0198; L-NAME: $p$=0.0314.
**(C)** mRFP-GFP-LC3 HeLa cells – L-NAME: $p$=0.0332.
**(D)** *Atg5*$^{+/+}$ MEFs – L-NAME: $p$<0.0001.
*Atg5*$^{-/-}$ MEFs – L-NAME: $p$=0.354; Control *Atg5*$^{+/+}$ MEFs vs Control *Atg5*$^{-/-}$ MEFs: $p$<0.0001.
**(E)** *Drosophila* Q120 + DMSO (Control; Mean ± SEM: 3.2 ± 0.3) vs *Drosophila* Q120 + L-NAME (Mean ± SEM: 4.4 ± 0.2): $p$<0.001 (paired $t$-test, based on n=4 independent sessions, 15 ommatidia scored from 30 flies per session).
**(F)** *Drosophila* Q120 (Control; Mean ± SEM: 3.7 ± 0.2) vs *Drosophila* Q120 + NOS4 (Mean ± SEM: 4.8 ± 0.5): $p$<0.05 (paired $t$-test, based on n=4 independent sessions, 15 ommatidia scored from 15 flies per session).
**(H)** HD zebrafish – Rapamycin: $p$<0.01; L-NAME: $p$<0.01.
**(I)** HD zebrafish – $NH_4Cl$: $p$<0.01; L-NAME: $p$<0.05; $NH_4Cl$ + L-NAME: $p$<0.01.
